# Host sex disparity and viral genotype dependence of the glycosylation level of small Hepatitis B surface protein in patients with HBeAg-positive chronic Hepatitis B

**DOI:** 10.1186/s12985-023-02096-x

**Published:** 2023-07-19

**Authors:** Guomin Ou, Chengyu Zhao, Juan Deng, Hui Zhuang, Kuanhui Xiang, Tong Li

**Affiliations:** Department of Microbiology and Infectious Disease Center, School of Basic Medical Sciences, Peking University Health Science Center, Beijing, 100191 China

**Keywords:** HBsAg, SHBs, Glycosylation, Genotype, Sex, Western blot

## Abstract

**Background:**

Hepatitis B surface antigen (HBsAg) consists of six components of large/middle/small HBs proteins (L/M/SHBs) with non-glycosylated (ng)- or glycosylated (g)- isomers at sN146 in their shared S domain. g-SHBs plays a crucial role in hepatitis B virus (HBV) secretion. However, the host and viral factors impacting sN146 status in natural HBV infection remain revealed mainly due to the technical difficulty in quantifying g-SHBs and ng-SHBs in serum samples.

**Methods:**

To establish a standardized Western blot (WB) assay (WB-HBs) for quantifying the SHBs isomers in serum samples of 328 untreated hepatitis B e antigen (HBeAg)-positive chronic hepatitis B (CHB) patients with genotype B or C HBV infection. The 1.3-mer HBV genotype B or C plasmids were transiently transfected into HepG2 cells for in vitro study.

**Results:**

The median level of ng-SHBs was significantly higher than that of g-SHBs (N = 328) (2.6 vs. 2.0 log_10_, *P* < 0.0001). The median g-/ng-SHBs ratio in female patients (N = 75) was significantly higher than that of male patients (N = 253) (0.35 vs. 0.31, *P* < 0.01) and the median g-/ng-SHBs ratio in genotype C patients (N = 203) was significantly higher than that of the genotype B patients (N = 125) (0.33 vs. 0.29, *P* < 0.0001).

**Conclusions:**

Our findings suggest that the g-/ng-SHBs ratio is host-sex-biased and viral genotype dependent in treatment naïve patients with HBeAg-positive chronic hepatitis B, which indicates the glycosylation of SHBs could be regulated by both host and viral factors. The change of ratio may reflect the fitness of HBV in patients, which deserves further investigation in a variety of cohorts such as patients with interferon or nucleos(t)ide analogues treatment.

**Supplementary Information:**

The online version contains supplementary material available at 10.1186/s12985-023-02096-x.

## Background

World Health Organization estimates that 296 million people worldwide are chronically infected with hepatitis B virus (HBV), who are at higher risks of liver cirrhosis and liver cancer [1, 2]. In addition to producing infectious Dane particles, HBV secretes large amounts of subviral particles (SVP) formed by hepatitis B surface antigen (HBsAg) [3]. HBsAg plays an important role in the viral life cycle, diagnosis and prognosis of chronic hepatitis B (CHB), and its clearance is a key indicator of functional cure of CHB [4]. However, this goal is difficult to achieve with current antiviral therapies, such as interferons and nucleos(t)ide analogues.

**Fig. 1:**
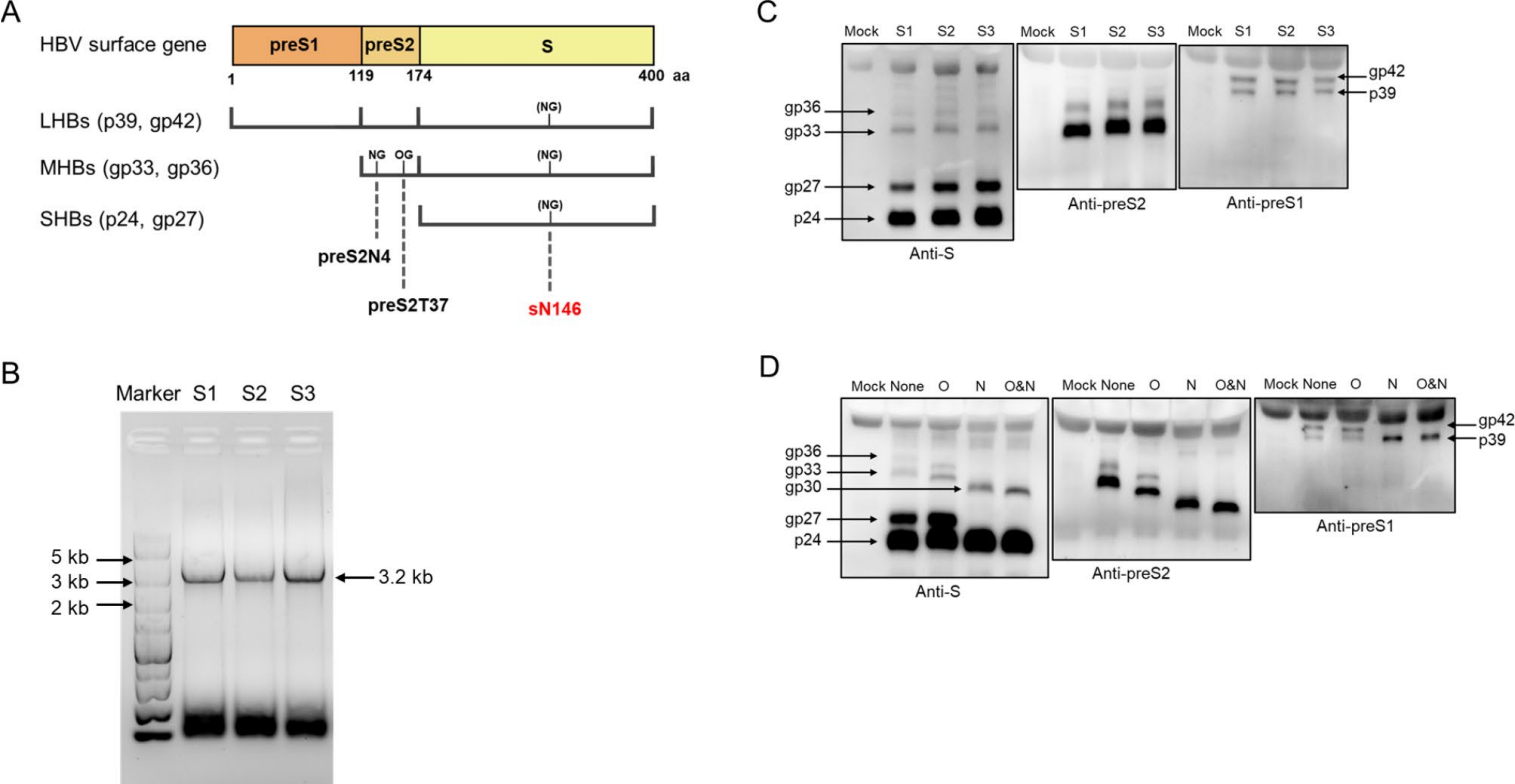
Diagrammatic representation of the L/M/SHBs and characterization of the serum samples as EC. **(A)** HBV surface gene consists of preS1, preS2 and S domains, which all or partially participate in construction of LHBs, MHBs and SHBs proteins. preS2N4 and preS2T37 in MHBs were N-glycosylated (NG) and O-glycosylated (OG), respectively. sN146 was proportionally N-glycosylated in LHBs, MHBs and SHBs proteins, which forms two-bands pattern in molecular weight (24 and 27 kD of SHBs; 33 and 36 kD of MHBs; 39 and 42 kD of LHBs). (**B**) Amplification of full-length HBV genome in three serum samples of S1-S3 by polymerase chain reaction (PCR). HBV DNA was extracted from 200 µl serum sample and then amplified with PrimeSTAR Max DNA Polymerase. The PCR products were analyzed by 1% agarose gel electrophoresis. The 3.2 kb of HBV genome bands were indicated. (**C**) L/M/SHBs of three patient serum samples (S1-S3) were detected by WB assay. Anti-S, anti-preS2, and anti-preS1 antibodies were used to detect proteins containing S, preS2 or preS1 domain, respectively. (**D**) PNGase F (N) was used to digest N-glycan. α2–3,6,8,9 Neuraminidase A combined with O-Glycosidase (O) were used to remove O-glycan. O&N removed both O- and N-glycans. After digestion with N, O or O&N, S3 was further examined by WB assay to confirm the correct positions of L/M/SHBs bands. The serum of HBV non-infected individual was as mock

HBsAg is composed of three hepatitis B surface proteins (HBs), namely large (L), middle (M), and small (S) HBs, which share the carboxyl terminal S domain. SHBs consists only of the S domain, MHBs is composed of S domain and its amino (N)-terminal extended preS2 domain, while MHBs and its N-terminal elongated preS1 domain constitute LHBs (Fig. 1A). SHBs is the most abundant HBs component of Dane particles and SVPs. Several studies have investigated the clinical significance of HBsAg composition. Pfefferkorn et al. found that the individuals at the inactive HBV carrier phase had significantly lower proportions of LHBs and MHBs than those in the patients at acute or chronic phases irrespective of their hepatitis B e antigen (HBeAg) status or HBsAg levels [5]. They also found that the median MHBs level at treatment baseline was significantly lower in patients with subsequent HBsAg loss than that in those without HBsAg loss after therapy [6]. However, Rinker et al. found that HBs levels differed by HBV genotype, and quantification of HBs species has no advantage over the commercial HBsAg assays for predicting response to peginterferon alfa-2a therapy in HBeAg-positive patients [7]. The biological and clinical significance of L/M/SHBs still needs more investigations.

In addition to the differences in the amino acid sequence and ratio of LHBs/MHBs/SHBs, HBs composition is further complicated by glycosylation modification (Fig. 1A). Complete N-linked glycosylation and O-linked glycosylation occur at the preS2 N4 and T37 positions of MHBs, respectively [8, 9]. Moreover, the sN146 is partially glycosylated, thus L-, M- and S- HBs each forms two protein isomers with different molecular weights, namely p39 and gp42, gp33 and gp36, p24 and gp27, respectively [10]. Glycosylation increases the diversity of protein structure and impacts the protein function. Many viral proteins, especially surface proteins, acquire glycans by employing host cell processing machinery. This modification impacts viral secretion, immunogenicity, infectivity and pathogenicity [11, 12]. HBV infection relies not only on the binding of the preS1 domain of LHBs to sodium taurocholate cotransporting polypeptide (NTCP) of human hepatocytes [13, 14], but also on the low affinity binding of antigenic loop [AGL, amino acid (AA) 102–161] of SHBs to heparan sulfate proteoglycan (HSPG) [15]. The AA 146 to 148 of SHBs (Asn-X-Ser/Thr, X is any amino acid except proline), a sequon essential for N-linked glycosylation, is just located within the AGL and is very conserved. N-linked glycosylation of SHBs is essential for HBV virion secretion, but is dispensable for secretion of HBV SVP and HDV [16, 17], which suggests that glycosylated N146 is specifically critical for the secretion of HBV virions. However, HBV is naturally packaged by glycosylated (g-) and non-glycosylated (ng-) SHBs that hints both isomers of SHBs are needed for HBV survival. Of note, some HBV virion secretion defects caused by mutations at non-sN146 site in the SHBs can be rescued by an extra N-glycosylation site [18]. Additionally, other studies have shown that additional N-linked glycosylation rather than at sN146 site in the major hydrophilic region (MHR, AA 99–169) of the SHBs could cause HBV immune escape [19, 20]. Anyhow, the N-glycosylation pattern of SHBs in patients’ sera as well as its biological and clinical implication are still waiting to be revealed.

Our hypothesis is that an appropriate ratio of g- to ng-SHBs (g-/ng-SHBs) might help maintain the ratio of HBV virion to SVP within a certain range, which should have an important virological significance and clinical consequence. However, the quantification of g- and ng-SHBs has yet been evaluated in patients’ sera, and the potential clinical significance of this ratio remains unclear largely due to the difficulties in specifically quantifying g- and ng-SHBs isomers among the complex host and viral protein components in serum. Thus, a standardized assay for quantifying SHBs isomers in serum is crucial for probing the implication of this ratio. Although SHBs isomers share identical AA sequences, they differ in molecular weight by about 3 kD due to sN146 glycosylation. This inspires us to attempt to establish a standardized assay based on the principle of Western blot (WB) for quantification of g- and ng-SHBs in serum (WB-HBs) to facilitate the study on g-/ng-SHBs ratio.

In this study, we first established such a WB-HBs assay, which was then used to characterize SHBs isomers and g-/ng-SHBs ratio in the serum samples of 328 untreated HBeAg-positive patients with genotype B (GTB) or genotype C (GTC) HBV infection. The results revealed that the glycosylation level of SHBs is characterized by host sex disparity and genotype dependence. The effect of HBV genotype on SHBs glycosylation was also verified by in vitro transfection experiments in HepG2 cell line. This paves the way for further understanding of this partially glycosylated SHBs.

## Methods

### Serum samples and standard laboratory assessment

The serum samples of 328 untreated HBeAg-positive Chinese CHB patients with GTB or GTC HBV infection were from our previous registered clinical study (NCT01088009) [21, 22]. The informed consents were obtained. The clinical diagnosis of CHB was performed according to the Chinese Society of Hepatology. Patients with hepatic decompensation or hepatocellular carcinoma were excluded according some criteria described in our previous study [22]. The serum samples were negative for hepatitis C virus, hepatitis D virus and human immunodeficiency virus serum markers and stored in aliquots at -80 ℃ until use.

The serum HBsAg level was detected by ARCHITECT HBsAg kit (Abbott Diagnostics, Chicago, United States) [22]. Serum level of HBV DNA was quantitated by TaqMan 48 automatic fluorescence quantitative PCR kits using Roche COBAS AmpliPrep/COBAS TaqMan 48 Analyzer (Roche Diagnostics, Mannheim, Germany) [22]. Serum level of HBV RNA was quantitated by our published in-house assay [21].

### WB-HBs assay for patient serum samples and HepG2 cells for validation of genotype effect

The WB-HBs assay was established based on external control (EC) and loading control (LC) for detecting the g- and ng- SHBs levels (for details see **Supplementary Material**). HBV GTB or GTC replicon plasmid was transfected into HepG2 cells for validating genotype dependence of SHBs glycosylation level (for details see **Supplementary Material**).

### Statistical analyses

All data were plotted using Origin 2022 software (OriginLab Corporation, Northampton, MA, United States). Relationships among continuous variables were assessed using Spearman’s rank correlation coefficients. Student’s t-test or Mann-Whitney U test was used to examine the difference for continuous variables and Chi-square test was used to analyze categorical variables. Univariate and multivariate logistic regression were used to determine independent factors associated with higher serum g-/ng-SHBs ratio in untreated HBeAg-positive CHB patients. Statistical analyses were performed using IBM SPSS statistics 19.0 software (IBM Corp., Armonk, NY, USA) and *P* < 0.05 was considered as statistically significant.

## Results

### Selection of EC sera and specificity confirmation of HBs protein bands

An EC is necessary for a comparable quantification of the SHBs bands on different PVDF membranes in a reliable WB-HBs assay. The 3.2 kb full-length HBV genomes of serum sample S1 to S3 were amplified by PCR and the products were resolved in the 1% agarose gel by electrophoresis and visualized by staining with Ultra GelRed Nucleic Acid Stain (Vazyme Biotech) (Fig. 1B), then preS/S region was sequenced by Sanger method. As shown in **Supplementary Table **S1, S1 to S3 met the EC criteria, i.e., no detectable indel, point mutations at glycosylation and well-known antigenicity-related sites within the preS/S region was detected [23–26]. Thus, all could be used as EC candidates.

After sequencing confirmation, the gene expression was further studied by subjecting S1 to S3 serum samples to WB assay. The anti-S, anti-preS2 and anti-preS1 antibody was used, respectively. As illustrated in Fig. 1A, the preS/S open reading frame (ORF) would produce three HBs species (L/M/SHBs), each of them would form two molecular weight proteins due to partial sN146 glycosylation. Moreover, it is known that preS2N4 and preS2T37 are N-glycosylated and O-glycosylated in MHBs instead of LHBs, respectively [8, 9]. The results shown in Fig. 1C indicated that the g- and ng-L/M/SHBs isomers had band patterns as expected and SHBs accounts for the largest proportion of HBsAg (about 68.1%). We further verified the identity of each band from sample S3 on PVDF membranes by glucosidase digestion. After O-glycosidase and α2–3,6,8,9 neuraminidase A (New England Biolabs, Ipswich, United States) digestion, the two suspected MHBs bands (33 and 36 kD) on PVDF membrane with different molecular weight moved faster, indicating they were O-glycosylated. After PNGase F (New England Biolabs) digestion, the 39/30/24 kD bands of L/M/SHBs were maintained, indicating that L/M/SHBs were separated into dual molecular weight bands due to N-linked glycosylation of sN146 (Fig. 1D). The results were consistent with literature reports [9, 10] and sequencing data (**Supplementary Table **S1). Thus, the S3 serum was used as an EC for the following experiments in this study, while the S1 and S2 sera were kept as potential ECs for the future cohort studies.

### Expression, purification and antigenicity of LC

**Fig. 2:**
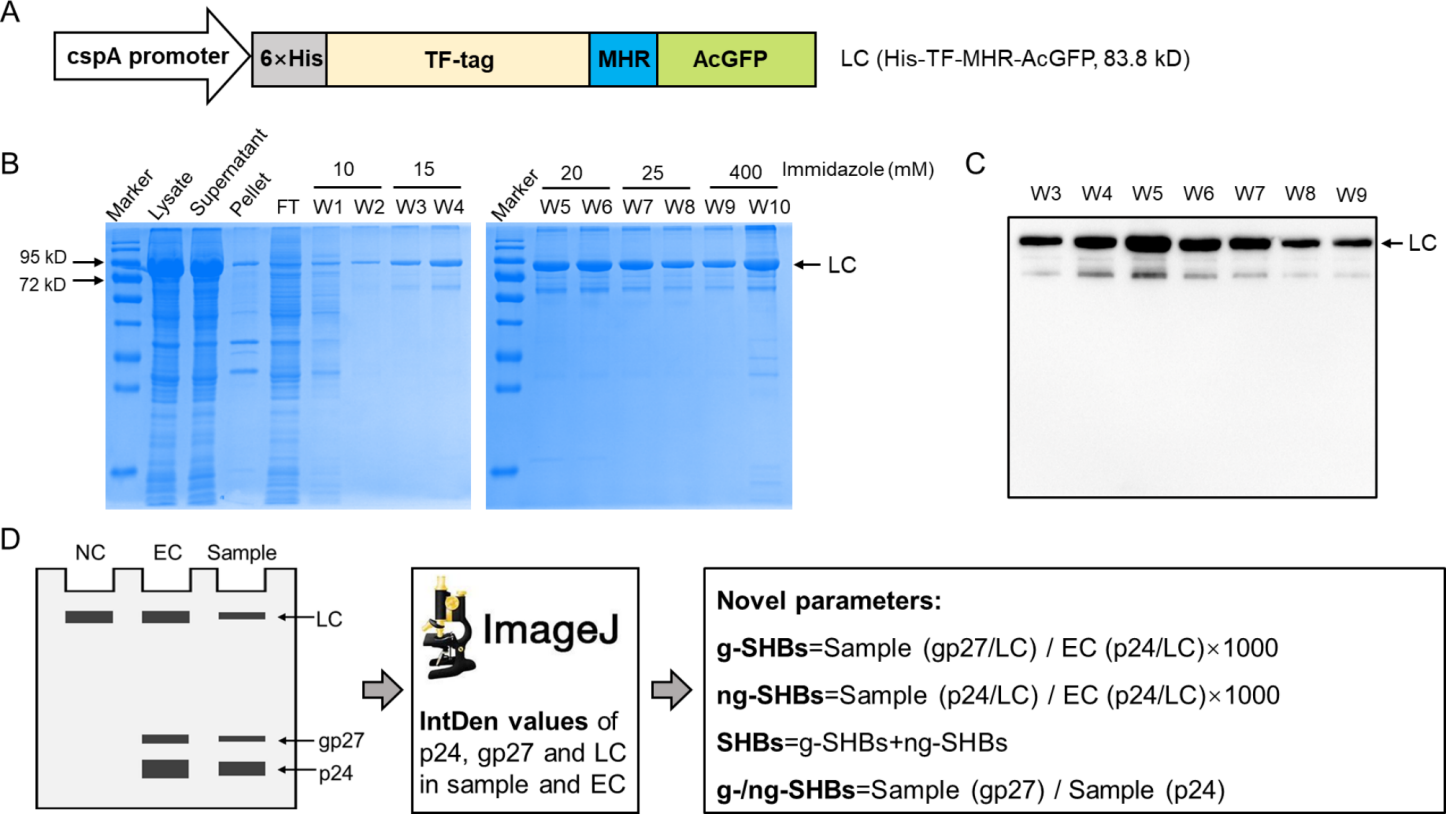
A loading control (LC) protein preparation for WB-HBs assay and the flow chart for SHBs novel parameters acquisition. (**A**) The recombinant protein His-TF-MHR-AcGFP, which was expressed as LC, is at the immediate downstream of cspA promoter in pCOLD-TF plasmid. The gray box indicates 6×His tag, which is sequentially fused by trigger factor (TF-tag), major hydrophilic region (MHR) and AcGFP protein. (**B**) LC is expressed in *E.coli* BL21 strain. The cell lysate post sonication was centrifugated at 10,000 g for 20 min at 4℃. The supernatant was subjected to column filled with Ni-NTA agarose beads in binding buffer. After flow through (FT), the beads were washed with two bed volumes binding buffer with imidazole concentration of 10, 15, 20, 25 and 400 mM (W) sequentially. All fractions were subjected to electrophoresis in 10% SDS-PAGE and stained with Coomassie blue for visualization. About 83.8 kD of LC is indicated by arrow. (**C**) LC were detected by WB assay with anti-S antibody in W3 to W9 of washed fractions. (**D**) Illustration of a WB-HBs assay, process of SHBs bands and SHBs novel parameters calculation. The serum of HBV non-infected individual served as negative control (NC). Serum sample S3 used as external control (EC) runs in each WB assay for comparing gray values (IntDen values in ImageJ software) of SHBs bands among different PVDF membranes. Before performing WB assay, NC, EC or Sample were mix with equal quantity of LC used for normalizing loading amounts. The InterDen value of each WB band (LC, p24 and p27) in EC or Samples were measured by ImageJ software. ng-SHBs, g-SHBs, SHBs and the g-/ng-SHBs ratio were calculated by functions as showed in (**D right**). EC, external control; g, glycosylation; LC, loading control; NC, negative control; ng, non-glycosylation; SHBs, small hepatitis B surface protein

In order to normalize the loading amount, a recombinant protein His-TF-MHR-AcGFP was constructed as a LC. As shown in Fig. 2A, the LC consists of four parts, namely 6×His, TF-tag, HBV MHR and AcGFP. The 6×His is used as a purification tag, TF-tag promotes solubility of recombinant protein, MHR contains a main antigenic epitope “a” determinant of HBsAg for anti-S antibody detection and AcGFP increases molecular weight of LC to help separate it from HBs species on SDS-PAGE. The cspA, a promoter of the cold-shock gene in *E. coli*, is used to robustly drive the expression of LC at 16℃. LC was expressed in *E. coli* BL21 strain and purified by Ni-NTA column as shown in Fig. 2B. Cells were harvested by centrifugation and lysed by sonication. A large amount of LC was expressed and mainly existed in the supernatant. Most of Ni-NTA bonded LC was eluted by washing buffer of W5 to W10 containing 20 to 400 mM imidazole (Fig. 2B). Washing fractions of W3 to W9 were subjected to WB assay and showed highly reactivity with anti-S antibody at about 84 kD position as predicted (Fig. 2C). The W3 to W9 fractions were harvested and combined as purified LC and quantified (7.2 µg/µl) by bicinchoninic acid protein assay.

### Establishment of a WB-HBs assay for quantification of g-SHBs and ng-SHBs in the serum samples

The flow chart of WB-HBs assay is shown in Fig. 2D. An EC, a negative control (NC) of HBV negative serum and the testing serum samples, each containing the LC, were loaded onto the SDS-PAGE. After WB-HBs assay, the gray values (IntDen values) of p24, gp27 and LC from EC and samples were measured by ImageJ software. The relative levels of SHBs, g-SHBs, ng-SHBs and g-/ng-SHBs were calculated by the functions displayed in the right panel of Fig. 2D.

The serum samples were pretreated using the procedure described in the Materials and Methods section. To determine the intra- and inter-assay precision of the quantification of g-SHBs and ng-SHBs, one serum sample was tested using 12 replicates in one WB-HBs run and one sample was tested in 15 independent WB runs, respectively. The intra-assay coefficient of variation (CV) of g-SHBs and ng-SHBs was 2.87% and 1.20%, respectively; while the inter-assay CV of g-SHBs and ng-SHBs was 2.39% and 1.39%, respectively (**Supplementary Table **S2).

### Characteristics of patients

The demographic, biochemical and virological characteristics of 328 HBeAg-positive CHB patients without treatment were summarized in Table 1. Most patients were young (median age: 30 years), male (77.1%), and infected with HBV GTB (38.1%) or GTC (61.9%). The median HBsAg, HBV DNA and HBV RNA levels were 4.3 log_10_ IU/ml (range: 2.8–5.5 log_10_ IU/ml), 8.0 log_10_ IU/ml (range: 4.1–9.8 log_10_ IU/ml) and 5.3 log_10_ IU/ml (range: 1.8–7.3 log_10_ IU/ml), respectively.

**Table 1:** Demographic, biochemical and virological characteristics of HBeAg-positive CHB patients without treatment

Characteristics	Patients (N = 328)
Sex, male/female (male%)	253/75 (77.1)
Age (years)^#^	30.0 (18.0–64.0)
ALT (U/L)^#^	119.0 (24.1–1150.0)
AST (U/L)^#^	69.9 (22.2–985.0)
HBsAg (log_10_ IU/ml)^#^	4.3 (2.8–5.5)
HBV DNA (log_10_ IU/ml)^#^	8.0 (4.1–9.8)
HBV RNA (log_10_ IU/ml)^#^	5.3 (1.8–7.3)
HBV genotype, n (%)	
B	125 (38.1)
C	203 (61.9)

ALT, alanine aminotransferase; AST, aspartate aminotransferase; CHB, chronic hepatitis B; HBeAg, hepatitis B e antigen; HBsAg, hepatitis B surface antigen; HBV, hepatitis B virus; ^#^, Median (range)

### Detection of ng-SHBs and g-SHBs in patient serum samples

**Fig. 3:**
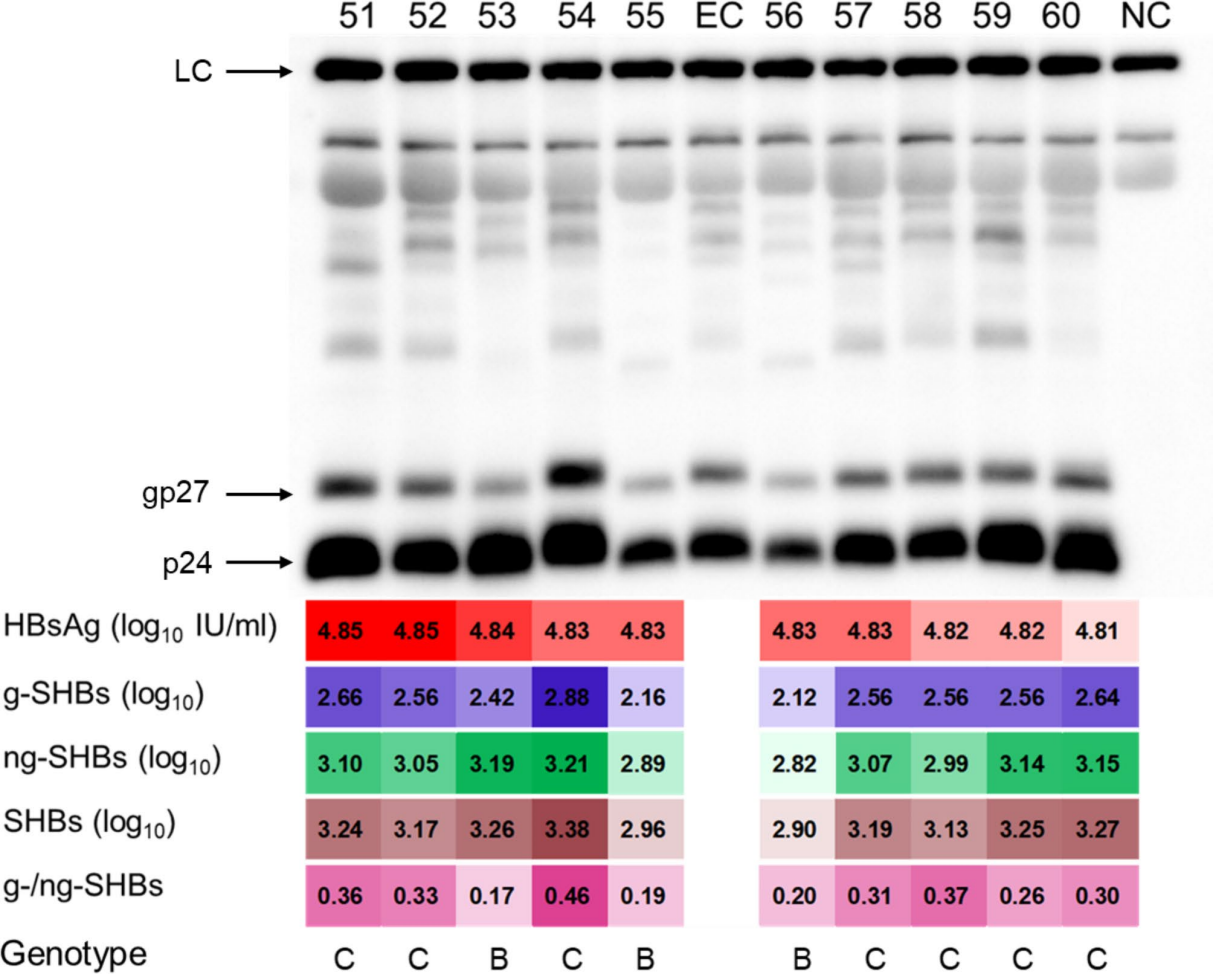
A representative picture from the WB-HBs assay. LC, p24 and gp27 were detected in EC and sample 51 to 60 by WB-HBs assay. Post calculation, the values of HBsAg and novel parameters were filled with different colors at the bottom of the picture and the intensity of colors depends on the size of values. EC, external control; g, glycosylation; HBsAg, hepatitis B surface antigen; LC, loading control; NC, negative control; ng, non-glycosylation; SHBs, small hepatitis B surface protein

Next, we used the established WB-HBs assay to analyze the SHBs isomers in serum samples from 328 patients. All testing samples, in which the lowest level of serum HBsAg was 2.8 log_10_ IU/ml (676.0 IU/ml), gave measurable signals. Figure 3 was a representative WB-HBs picture showing the results of sera No. 51 to 60, which were arranged in the order of high to low serum HBsAg levels (70584.0 IU/ml to 65188.0 IU/ml) measured by ARCHITECT HBsAg kit. It was obvious that the quantitative HBsAg levels were not exactly consistent with the levels of SHBs components assayed by the WB-HBs (Fig. 3), which could be partly due to the fact that HBsAg is the sum of three proteins: SHBs, MHBs and LHBs.

**Fig. 4:**
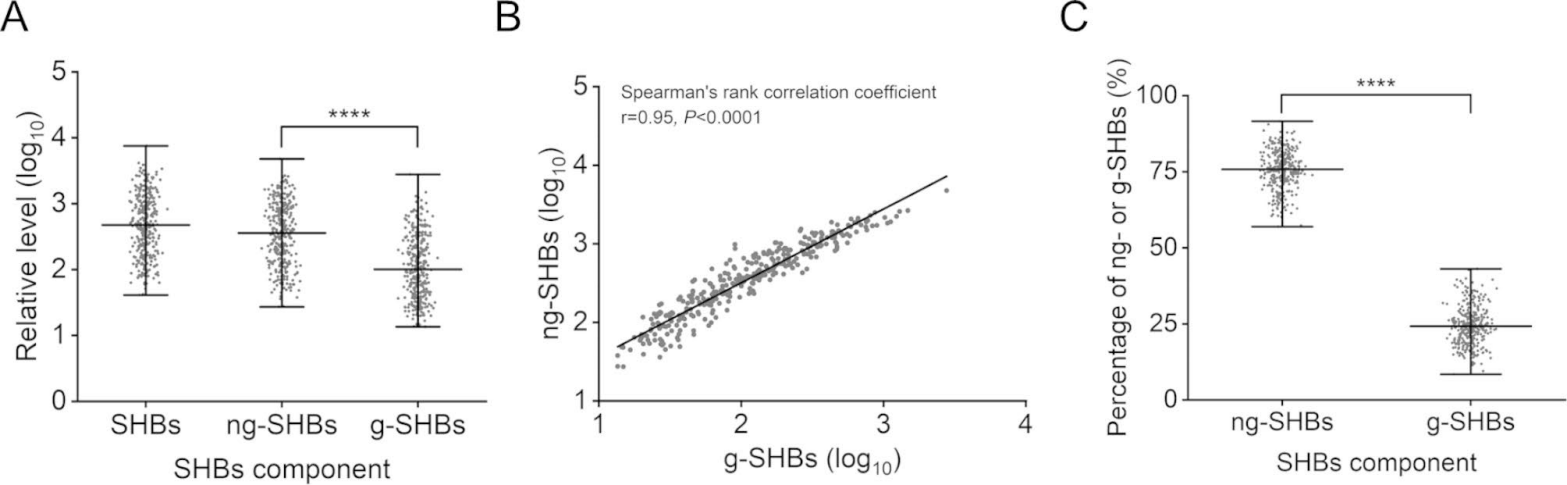
SHBs protein levels (**A**), Spearman’s rank correlation coefficient between g-SHBs and ng-SHBs levels (**B**), and SHBs proportions (**C**) in treatment-naïve patients with HBeAg-positive chronic hepatitis B. The *P* value was determined using Mann-Whitney U test; ****, *P* < 0.0001; g, glycosylation; ng, non-glycosylation; SHBs, small hepatitis B surface protein

Four novel parameters for SHBs components of the testing samples were summarized in Table 2. The median values of SHBs, ng-SHBs, g-SHBs levels were 2.7, 2.6 and 2.0 log_10_ with the difference between the extremes of 2.3 log_10_ each, respectively. The median level of ng-SHBs was significantly higher than that of g-SHBs (2.6 vs. 2.0 log_10_, *P* < 0.0001) (Fig. 4A), and these two parameters displayed a very strong positive linear correlation (r = 0.95, *P* < 0.0001) (Fig. 4B). Regarding the g-/ng-SHBs ratio, the median value was 0.32, but the range (0.09 to 0.76) was very large, suggesting the glycosylation level had a significant individual variation. In general, the median percentage of ng-SHBs/SHBs was about three times higher than that of g-SHBs/SHBs (75.9% vs. 24.2%, *P* < 0.0001) (Fig. 4C).

**Table 2:** Novel SHBs parameters of HBeAg-positive CHB patients without treatment

Parameter^#^	Patients (N = 328)
SHBs (log_10_)	2.7 (1.6–3.9)
ng-SHBs (log_10_)	2.6 (1.4–3.7)
g-SHBs (log_10_)	2.0 (1.1–3.4)
g-/ng-SHBs	0.32 (0.09–0.76)

CHB, chronic hepatitis B; g-, glycosylated; HBeAg, hepatitis B e antigen; ng-, non-glycosylated; SHBs, small hepatitis B surface protein; ^#^, Median (range)

### Correlation analyses of serum SHBs composition with host and viral factors

**Fig. 5:**
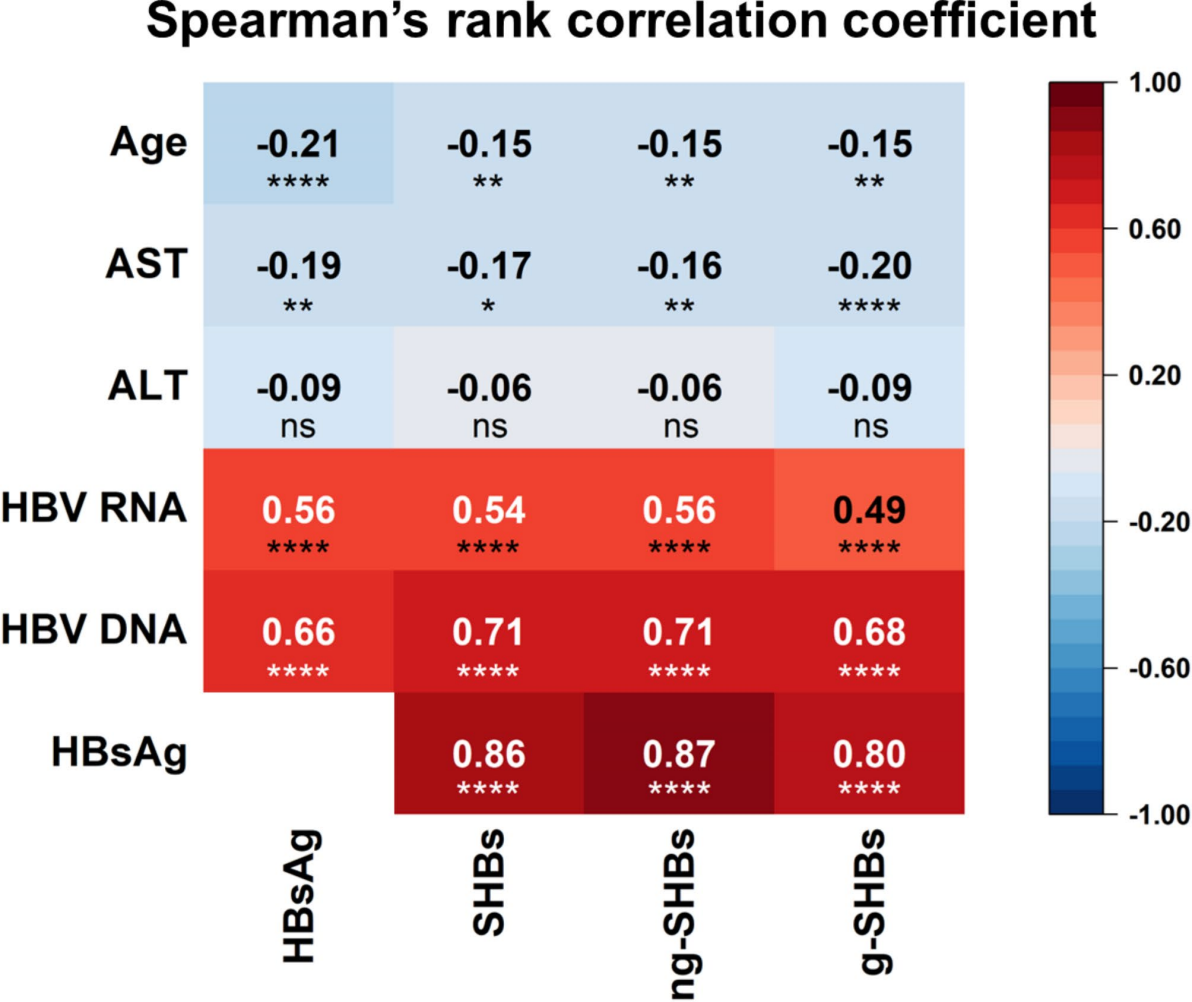
Correlation of disease parameters with SHBs protein levels. Spearman’s rank correlation coefficients were calculated; ns, no significant; *, *P* < 0.05; **, *P* < 0.01; ****, *P* < 0.0001; AST, aspartate aminotransferase; ALT, alanine aminotransferase; g, glycosylation; HBsAg, hepatitis B surface antigen; HBV, hepatitis B virus; ng, non-glycosylation; SHBs, small hepatitis B surface protein

As shown in Fig. 5, the levels of SHBs, ng-SHBs and g-SHBs displayed a strong positive linear correlation with serum HBsAg levels (r = 0.86, 0.87 and 0.80; all *P* < 0.0001) and HBV DNA (r = 0.71, 0.71 and 0.68; all *P* < 0.0001), and a moderate positive linear correlation with HBV RNA levels (r = 0.54, 0.56 and 0.49; all *P* < 0.0001), and a weak negative linear correlation with AST levels (r=-0.17, -0.16 and − 0.20, all *P* < 0.05) and ages (r=-0.15, -0.15 and − 0.15, all *P* < 0.01). However, no correlation between the ALT levels with each of the SHBs, ng-SHBs and g-SHBs markers was found.

### Potential impact factors of serum g-/ng-SHBs ratio

In this study, we used the serum g-/ng-SHBs ratio to reflect the glycosylation level of SHBs. This ratio had a significant individual variation and did not have strong and moderate linear correlation with any of the host and viral markers of age, AST, ALT, HBV DNA, HBV RNA and HBsAg (data not shown). To figure out the potential impacting factors, we classified patients into two groups based on the median value of g-/ng-SHBs ratio, namely low ratio group (LRG: ≤0.32) and high ratio group (HRG: >0.32), and performed univariate and multivariate logistic regression analyses to identify host and viral factors associated with higher serum g-/ng-SHBs ratio (Table 3). Female, lower AST level and HBV genotype C were independently associated with a higher g-/ng-SHBs ratio by univariate (all *P* < 0.05) and multivariate analysis (all *P* < 0.05) (Table 3). Univariate analysis also showed that lower HBV RNA level was associated with higher g-/ng-SHBs ratio (*P* < 0.05); however, this association was not apparent following multivariate analysis (Table 3). Moreover, the female and HBV genotype C displayed significant higher odds ratio, which appeared as the most interesting host and viral factors worthy for further investigation.

**Table 3:** Regression analysis of factors associated with higher g-/ng-SHBs ratio in untreated HBeAg-positive CHB patients (N = 328)

Parameter	Univariate			Multivariate		
	OR	95% CI	*P* value	OR	95% CI	*P* value
Female	1.815	1.072–3.072	**0.026**	1.900	1.077–3.350	**0.027**
Age (years)	1.019	0.995–1.044	0.129			
ALT (U/L)	0.998	0.996–1.000	**0.048**	1.002	0.998–1.006	0.306
AST (U/L)	0.996	0.993–0.999	**0.012**	0.993	0.987–0.999	**0.018**
HBsAg (log_10_ IU/ml)	0.678	0.449–1.025	0.065			
HBV DNA (log_10_ IU/ml)	1.025	0.778–1.350	0.861			
HBV RNA (log_10_ IU/ml)	0.813	0.677–0.977	**0.027**	0.911	0.752–1.104	0.341
HBV genotype C	2.139	1.357–3.372	**0.001**	2.181	1.353–3.514	**0.001**

ALT, alanine aminotransferase; AST, aspartate aminotransferase; CI, confidence interval; HBsAg, hepatitis B surface antigen; HBV, hepatitis B virus; OR, odds ratio

### SHBs composition in male and female patients with HBV infection

The patients were grouped by sex. The results in Table 4 indicated that the host factors (age, ALT and AST) as well as the HBV genotypes between the male and female patient groups had no significant difference, while the HBV DNA, HBV RNA, HBsAg, total SHBs and ng-SHBs levels were significantly higher in the male patients as compared with those in the female patients (*P* < 0.05), respectively. However, the g-SHBs had no significant difference between two groups (*P* > 0.05), resulting in significantly lower g-/ng-SHBs ratio in male patients than that in female patients (0.31 vs. 0.35, *P* < 0.01). It was found that female patients had higher frequency falling into the HRG than that of male patients (61.3% vs. 46.6%, *P* < 0.05) (**Supplementary Figure **S1).

**Table 4:** Clinical and virological characteristics in male or female patient groups

Characteristics	Sex		*P*-value
	Male (N = 253)	Female (N = 75)	
Age (years)^#^	29.0 (18.0–59.0)	31.0 (18.0–64.0)	0.419
ALT (U/L)^#^	125.0 (24.1–663.0)	102.0 (24.7–1150.0)	0.222
AST (U/L)^#^	66.0 (25.0–433.0)	75.5 (22.2–985.0)	0.206
HBV genotype, B/C (B%)	103/150 (40.7)	22/53 (29.3)	0.075
HBV DNA (log_10_ IU/ml)^#^	8.1 (4.1–9.8)	7.8 (5.4–9.2)	**0.020**
HBV RNA (log_10_ IU/ml)^#^	5.4 (1.8–7.3)	5.1 (1.8–7.1)	**0.011**
HBsAg (log_10_ IU/ml)^#^	4.4 (2.8–5.5)	4.2 (3.2–5.4)	**0.014**
SHBs (log_10_)^#^	2.7 (1.6–3.9)	2.5 (1.8–3.5)	**0.025**
ng-SHBs (log_10_)^#^	2.6 (1.4–3.7)	2.4 (1.6–3.3)	**0.011**
g-SHBs (log_10_)^#^	2.0 (1.1–3.4)	1.9 (1.2–3.1)	0.214
g-/ng-SHBs^#^	0.31 (0.09–0.76)	0.35 (0.21–0.66)	**0.001**

ALT, alanine aminotransferase; AST, aspartate aminotransferase; g-, glycosylated; HBsAg, hepatitis B surface antigen; HBV, hepatitis B virus; ng-, non-glycosylated; SHBs, small hepatitis B surface protein; ^#^, Median (range)

### SHBs composition in GTB and GTC HBV infection

To investigate the possible effect of HBV genotypes on the SHBs components, the patients were divided into GTB and GTC groups as shown in Table 5. The levels of HBV RNA and HBsAg in GTB patients were significantly higher than those in GTC patients, while total, ng- and g- SHBs showed no difference between two groups. Unexpectedly, the GTC patients had significantly higher levels of g-/ng-SHBs ratio as compared with those in GTB patients (0.33 vs. 0.29, *P* < 0.001). This could also be observed in Fig. 3, where the serum samples No. 53, 55 and 56 were from GTB infected patients and showed lower g-/ng-SHBs ratio than those of GTC samples. Further analysis found that GTC patients had higher frequency falling into the HRG than that of GTB patients (57.1% vs. 38.4%, *P* < 0.01) (**Supplementary Figure **S2).

**Table 5:** Clinical and virological characteristics in patient groups with HBV genotype B or C infections

Characteristics	Genotype		*P*-value
	B (N = 125)	C (N = 203)	
Sex, male/female (male%)	103/22 (82.4)	150/53 (73.9)	0.075
Age (years)^#^	28.0 (19.0–47.0)	31.0 (18.0–64.0)	**< 0.001**
ALT (U/L)^#^	122.0 (40.0–393.0)	117.0 (24.1–1150.0)	0.978
AST (U/L)^#^	65.0 (25.5–249.0)	73.0 (22.2–985.0)	0.694
HBV DNA (log_10_ IU/ml)^#^	8.0 (5.3–9.8)	8.0 (4.1–9.8)	0.499
HBV RNA (log_10_ IU/ml)^#^	5.6 (1.8–7.1)	5.3 (1.8–7.3)	**0.001**
HBsAg (log_10_ IU/ml)^#^	4.5 (3.1–5.5)	4.2 (2.8–5.5)	**< 0.001**
SHBs (log_10_)^#^	2.7 (1.6–3.5)	2.6 (1.6–3.9)	0.274
ng-SHBs (log_10_)^#^	2.6 (1.4–3.4)	2.5 (1.4–3.7)	0.161
g-SHBs (log_10_)^#^	2.1 (1.2–2.9)	2.0 (1.1–3.4)	0.740
g-/ng-SHBs^#^	0.29 (0.13–0.66)	0.33 (0.09–0.76)	**< 0.001**

ALT, alanine aminotransferase; AST, aspartate aminotransferase; g-, glycosylated; HBsAg, hepatitis B surface antigen; HBV, hepatitis B virus; ng-, non-glycosylated; SHBs, small hepatitis B surface protein; ^#^, Median (range)

**Fig. 6:**
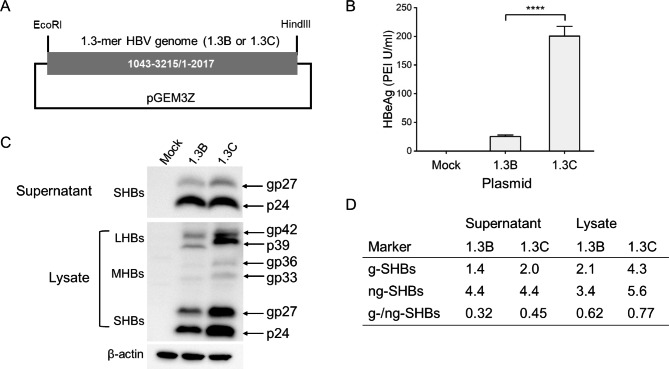
The level of g-/ng-SHBs ratio in HBV genotype (GT) C is higher than that in GTB in vitro. (**A**) 1.3-mer HBV genomes of GTB (1.3B) and GTC (1.3C) were cloned into pGEM3Z plasmid (see **Materials and Methods** for detailed description). HepG2 cells were transfected with 1.3B, 1.3C or pGEM3Z mock vector in 24-well or 6-well plate. At 48 h post transfection, the supernatants from 24-well plate were harvested for HBeAg (**B**) detection by chemiluminescence immunoassay, and precipitation of HBV associated particles by adding polyethylene glycol 8000 to 500 µl supernatants from 6-well plate to a final concentration of 12.5% was followed by incubation at 4℃ for overnight. The precipitates were collected by centrifugation at 10,000 g for 10 min and dissolved in 100 µl PBS. HepG2 cells of 6-well plate were lysed with 200 µl RIPA buffer. (**C**) The dissolved precipitates and cell lysates were subjected to Western blot assay. (**D**) The IntDen values of g- and ng- SHBs in supernatants and lysates were measured by ImageJ software and the levels of g-/ng-SHB ratio were calculated. The *P* value was determined using Student’s t-test; ****, *P* < 0.0001

In order to confirm the effect of viral genotype on g-/ng-SHBs ratio in vitro, we constructed plasmids containing 1.3-mer HBV genome of GTB (1.3B) and GTC (1.3C) as illustrated in (Fig. 6A). At 48 h post transfection of 1.3B or 1.3C in HepG2 cells, HBeAg levels in supernatants as a marker of HBV replication were detected by chemiluminescence immunoassay. The 1.3C had significantly higher HBeAg level as compared with that in 1.3B (Fig. 6B). The PEG8000-precipitated HBV associated particles of supernatants and cell lysates were subjected to WB-HBs assay with anti-S antibody (Fig. 6C). The IntDen values of g- and ng- SHBs were measured and their ratio were calculated (Fig. 6D). The g-/ng-SHBs ratios in the 1.3C were higher than those of the 1.3B irrespective of supernatants (0.45 vs. 0.32) or lysates (0.77 vs. 0.62) (Fig. 6D). For further validation, another set of GTB and GTC 1.3-mer plasmids (1.3b and 1.3c) were also tested. The Western blot analysis gave the similar results (**Supplementary Figure S3**) as found in the Fig. 6. The in vitro result was in line with the findings in serum samples.

## Discussion

Glycoprotein plays vital roles in multiple biological processes, such as protein folding, stability, intracellular transport, extracellular secretion, molecular recognition and interaction, which affect the assembly, secretion and infection in the viral life cycle [11, 12]. However, little is known about SHBs glycosylation in CHB patients because of methodological limitations. In the present study, a standardized WB-HBs assay was established, validated and used to test g-SHBs and ng-SHBs in the sera of 328 untreated HBeAg-positive CHB patients with GTB or GTC HBV infection. The derived parameter of g-/ng-SHBs ratio was then analyzed to uncover the glycosylation level and its influencing factors.

Initial effort was put on to develop a standardized WB assay that we named as WB-HBs for reliably and reproduciblely analyzing the SHBs isomers in the serum samples. To achieve this goal, a LC and an EC were developed; PEG8000 precipitation of serum was carried out before sample loading; a polyclonal antibody against the S domain of HBsAg was used for detection; and the acquisition of the gray values of the target protein bands and data processing were all standardized. In principle, the WB-based assay could distinguish protein isomers and avoid the interference by the antigen-antibody complex of target proteins, which ELISA-based assay has. As the results, the WB-HBs assay showed intra- and inter-assay CVs lower than 3%, measurable signal for all testing samples with HBsAg levels ranging from 676.0 IU/ml (2.8 log_10_ IU/ml) to 314,900 IU/ml (5.5 log_10_ IU/ml) that included eight samples of HBsAg/anti-HBs double-positive. In addition, the SHBs isomers quantified by WB-HBs assay showed significant positive linear correlations with the serum HBsAg levels measured by Abbott ARCHITECT HBsAg kit, as well as the serum HBV DNA and RNA levels (Fig. 5). In overall, we validated the reproducibility and accuracy of the novel WB-HBs assay for testing SHBs isomers in serum samples. The results also suggest the applicability of this assay for analyzing various serum samples, such as those from the patients with negative HBeAg or under antiviral treatment, etc.

To test the 328 serum samples using the validated WB-HBs assay showed that all samples displayed ng-SHBs and g-SHBs two bands pattern on PVDF membranes, and that of ng-SHBs was stronger than that of g-SHBs in every sample. The serum level of ng-SHBs was higher than that of g-SHBs and the difference was statistically significant (*P* < 0.0001) (Fig. 4A). These suggest that both isomers of SHBs are indispensable for HBV survival in chronic infection. However, we noticed that the g-/ng-SHBs ratio had about 8-fold difference (0.09 to 0.76) among patients (Table 2) and no mutation was detected at sN146 site by population-based sequencing of SHBs in 328 serum samples [22]. These findings indicate the mutations flanked sN146 could influence the SHBs glycosylation level, which deserve further study. The degree of glycosylation is not only related to the sequence and structure of the targeting protein that is going to be glycosylated, but also depends on the physiological state of the host and the activity of the glycosylation processing machinery within the cells [27]. In this sense, the g-/ng-SHBs ratio might reflect the HBV fitness at a certain host condition. To figure out the potential factors affecting serum g-/ng-SHBs ratio is of biological and clinical interest.

Of note, our results indicated that the GTC-infected female patients with lower AST level tended to have a significantly higher g-/ng-SHBs ratio than that of GTB-infected male patients with higher AST level (Table 3). About host factors, the liver is a major organ for the synthesis of glycoproteins. It is not surprising that the elevated AST reflecting severe liver injury might lead to a decreased glycosylation capacity, thus correlate to a lower g-/ng-SHBs ratio. In addition, the sex disparity of HBV-related liver diseases has long been noticed, which could be attributed to sex hormone effects other than gender behaviors or environmental impact [28]. Our observation of the higher g-/ng-SHBs ratios in females than in males (Table 4) suggests that the abundance and/or types of N-linked glycosylation enzymes may differ according to sex, and provides another experimental evidence for sex disparity of HBV infection. Sex differences in glycosylation have been reported in a variety of organisms including humans, mice, nematodes, insects and etc [29–31], which would cause changes in the glycosylation of the viruses that infect them.

Virus genotype, like a person’s sex, is the essential difference between virus strains. Comparing to GTB HBV, the association of GTC infection with higher g-/ng-SHBs ratios was found in patients (Table 3**and** Table 5) and confirmed in the in vitro experiments (Fig. 6). The difference of SHBs AA sequences between genotype B and C may affect the accessibility of N-linked glycosylation motif and lead to different g-/ng-SHBs ratio. It is worth noting that the AGL sequences of the GTB and GTC differ at least by three AAs (GTB/GTC: sT/I126, sT/S143 and sK/R160), which are genotype-dependent polymorphic AA sites. The sK/R160 is known as a HBV serotype related AA site, whereas the mutations at s126 site have been extensively studied and shown to affect HBV secretion or HBsAg antigenicity [32]. Whether they have any effect on the glycosylation of SHBs remains uncovered. Moreover, the differences in HBV replication and protein expression levels between GTB and GTC may impact the expression of N-linked glycosylation enzymes in the hepatocytes, which also need more investigation to reveal.

This study has some limitations. Our WB-HBs is a relative quantitative assay because the absolute quantity of the EC was not available at present. Although this may have little affection on the comparison of SHBs isomer levels and the derived parameter of g-/ng-SHBs ratio among patients, the absolute quantification data would be more informative. Considering the antibody specificity, an antibody binding the conserved sequences (excluding the sN146 site) of S protein from different genotypes would be a good choice. Unfortunately, we did not find such one. According to the instruction of the manufacturer, the antibody (Abcam ab9193) used in this study is generated from a highly purified Ad/Ay antigen that is contained in HBV genotype A to J [33]. This antibody has been cited by about 30 publications including studies on genotype A to D (https://www.abcam.com/products/primary-antibodies/hepatitis-b-virus-surface-antigen-aday-antibody-ab9193.html). Besides, we consider using the fully automated high-throughput WB system to improve the throughput of the assay in future since the manual work is very laborious [34, 35]. Although the proportion of MHBs and LHBs have been linked with some clinical significance [5, 6], these bands are not clearly detectable in most samples due to insufficient sensitivity of WB-HBs.

## Conclusions

In conclusion, our study measured the relative levels of g- and ng- SHBs in HBeAg-positive untreated CHB patients by the established novel WB-HBs assay. The female sex and HBV GTC were identified as the potential factors to confer higher degree of SHBs glycosylation. To explore the potential clinical significance of SHBs isomers, a variety of cohorts such as interferon or/and nucleos(t)ide analogue treatment patients used for further research is warrant. The results also remind us that the degree of glycosylation should be considered in the future in vitro study other than just comparing the presence or absence of glycosylation for better understanding its virological impacts.

## Electronic supplementary material

Below is the link to the electronic supplementary material.

Supplementary Material 1

Supplementary Material 2

## Data Availability

The datasets used and/or analyzed during the current study are available from. the corresponding author on reasonable request.

## Abbreviations

1.3B: Plasmid containing 1.3-mer HBV genome of GTB
1.3C: Plasmid containing 1.3-mer HBV genome of GTC
AGL: Antigenic loop
CHB: Chronic hepatitis B
EC: External control
g: Glycosylated
HBeAg: Hepatitis B e antigen
HBs: Hepatitis B surface protein
HBsAg: Hepatitis B surface antigen
HBV: Hepatitis B virus
LC: Loading control
MHR: Major hydrophilic region
NC: Negative control
ng: Non-glycosylated
PEG8000: Polyethylene glycol 8000
SVP: Subviral particles
TF: Trigger factor
WB-HBs: A standardized modified Western blot (WB) assay for quantification of g- and ng-SHBs
