# Supplementary material for: Host sex disparity and viral genotype dependence of the glycosylation level of small Hepatitis B surface protein in patients with HBeAg-positive chronic Hepatitis B
Source: Virol J. 2023 Jul 19;20:159. doi: 10.1186/s12985-023-02096-x (PMC10357594; doi:10.1186/s12985-023-02096-x)
Supplement: Supplementary file 1 — Supplementary Material 1 [file 12985_2023_2096_MOESM1_ESM.pdf]

Supplementary materials of the full images of gels and blots

Host sex disparity and viral genotype dependence of the glycosylation level of small hepatitis B surface protein in patients with HBeAg-positive chronic hepatitis B

Guomin Ou, Chengyu Zhao, Juan Deng, Hui Zhuang*, Kuanhui Xiang*, Tong Li*

Department of Microbiology and Infectious Disease Center, School of Basic Medical Sciences, Peking University Health Science Center, Beijing 100191, China

*Correspondence:
Hui Zhuang
zhuangbmu@126.com
Kuanhui Xiang
kxiang@bjmu.edu.cn
Tong Li
toglii97@bjmu.edu.cn

Figure 1B

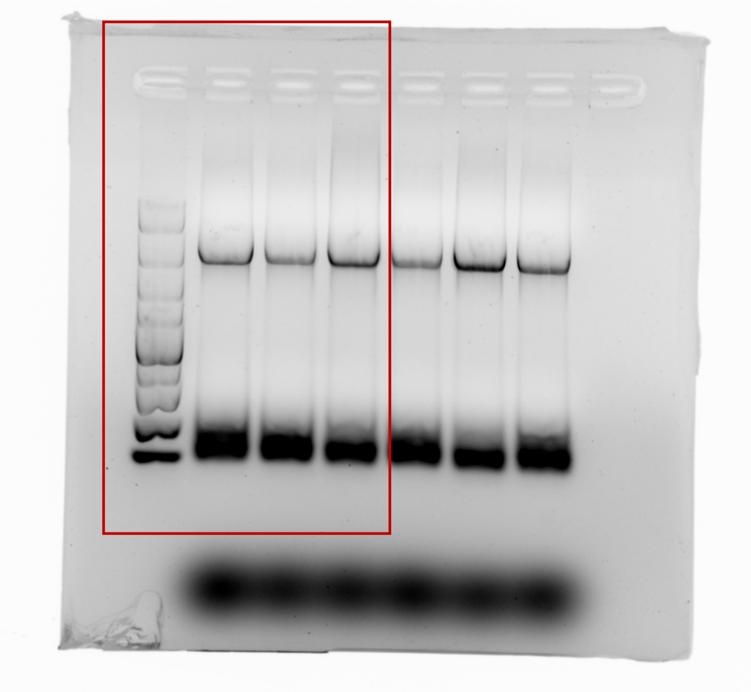

Agarose gel electrophoresis image used for the **Figure 1B**. The red rectangular box indicates the part presented in the paper.

Figure 1C left

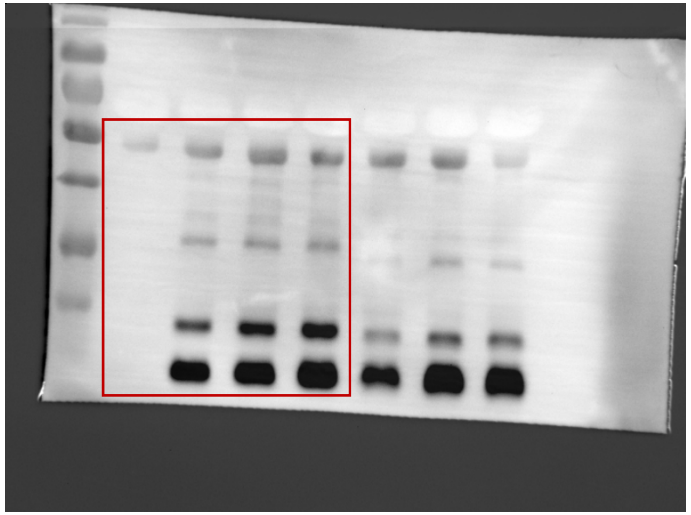

Figure 1C middle

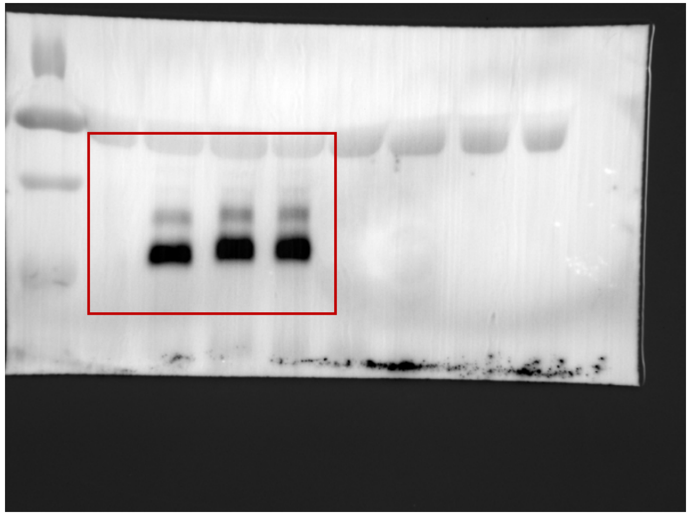

Figure 1C right

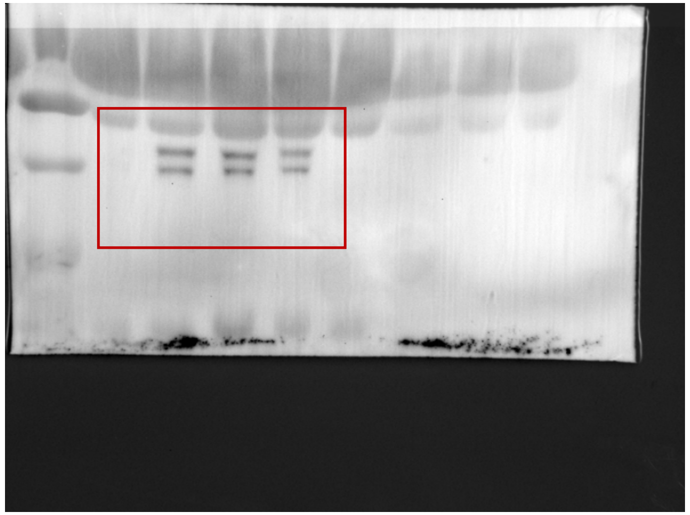

Western blot images used for the **Figure 1C**. The red rectangular box indicates the parts presented in the paper.

Figure 1D left

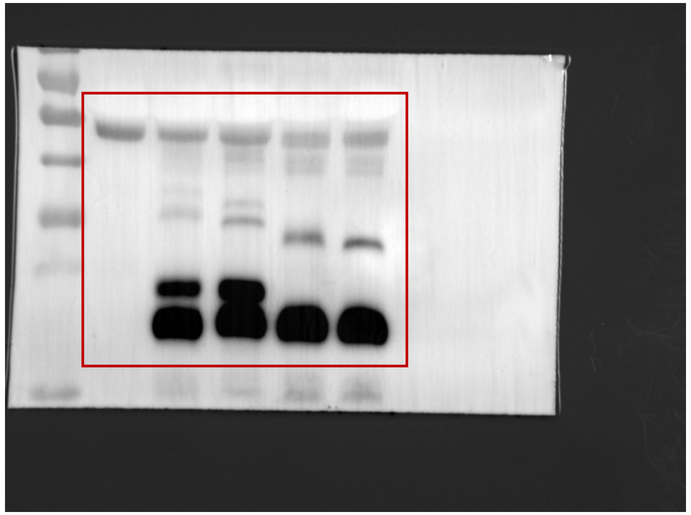

Figure 1D middle

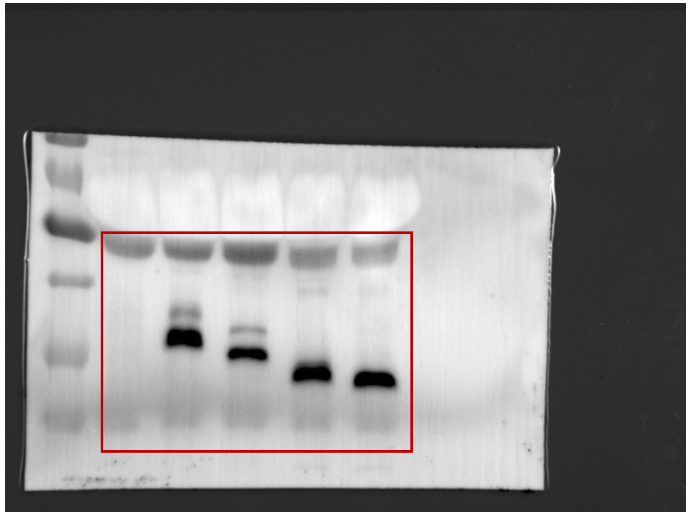

Figure 1D right

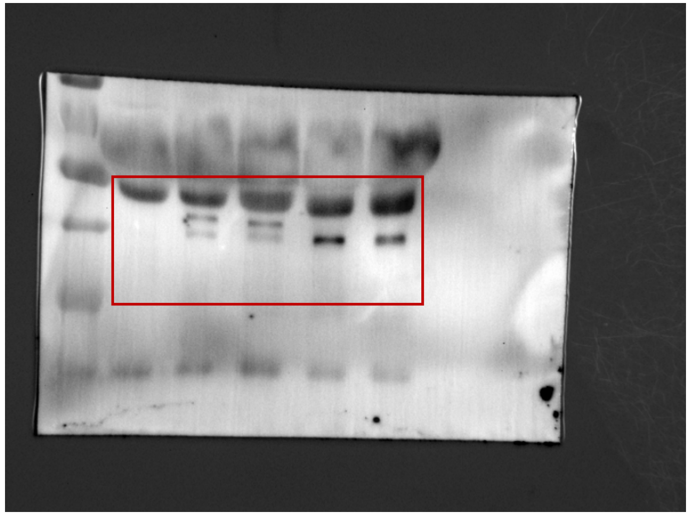

Western blot images used for the **Figure 1D**. The red rectangular box indicates the parts presented in the paper.

Figure 2B left

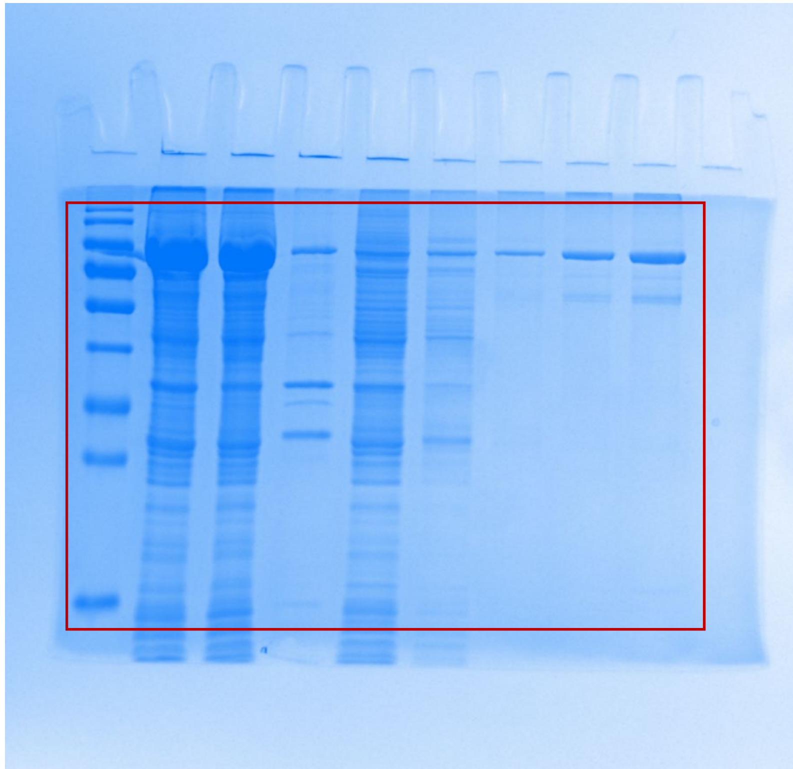

SDS-PAGE

Figure 2B right

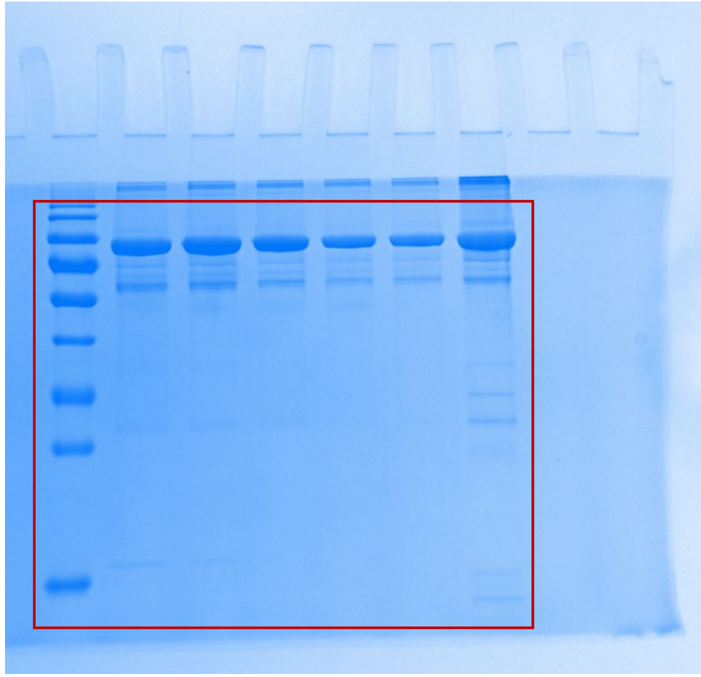

SDS-PAGE

Figure 2C

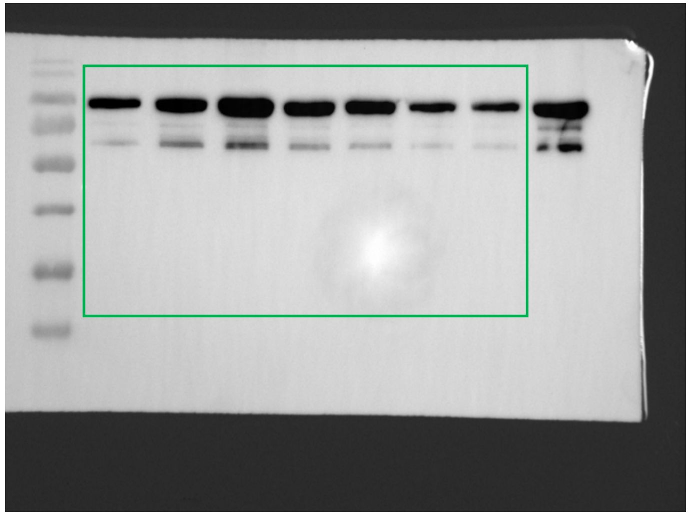

Western blot

The red and green rectangular boxes indicate the parts presented in the **Figure 2B and 2C**, respectively.

The pages 7 to 12 contain the Western blot images for the serum samples of patients, negative control (NC) and external control (EC), in which the target SHBs band intensities were measured and statistically analyzed. The data presented in the **Table 2**, **Table 4** and **Table 5** are derived from these images. The image named “Sample 51-60” on page 6 in this file is used as an example and presented in the **Figure 3** of this paper.

Sample 1-10

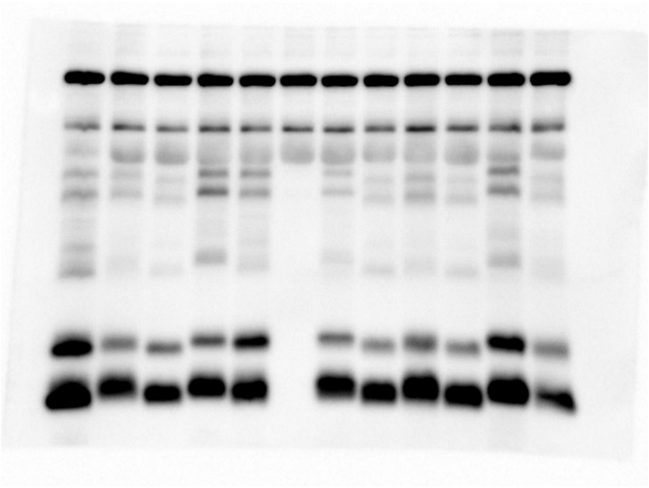

Sample 11-20

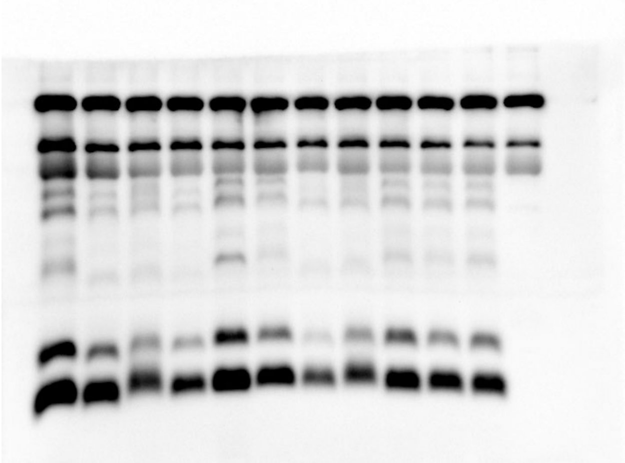

Sample 21-30

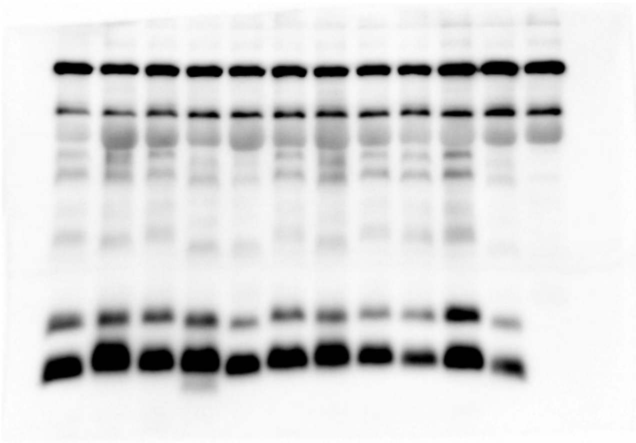

Sample 31-40

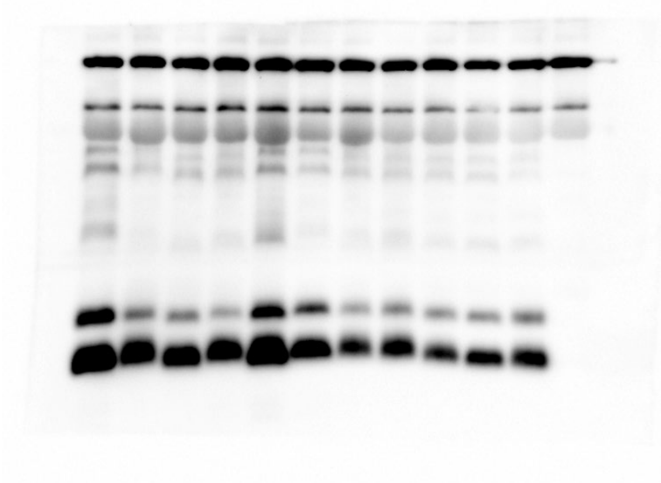

Sample 41-50

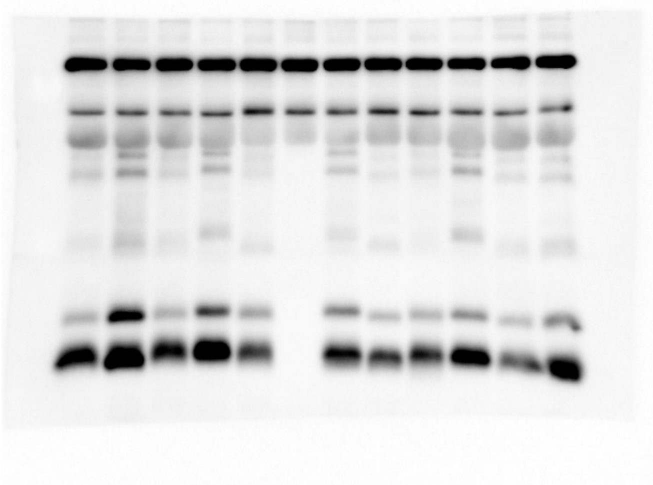

Sample 51-60

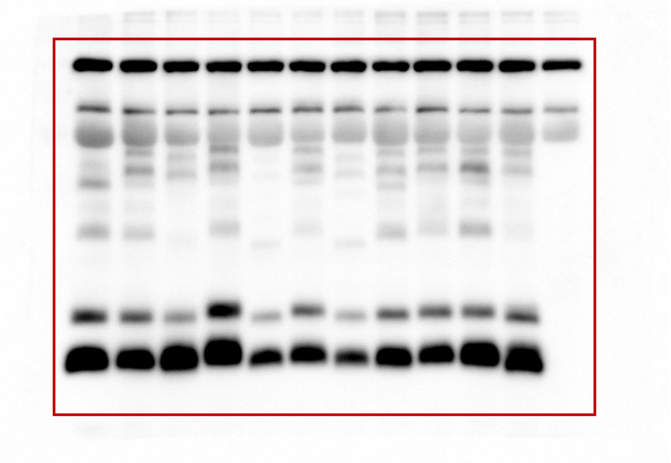

The red rectangular box indicates the parts presented in **Figure 3**.

Sample 61-70

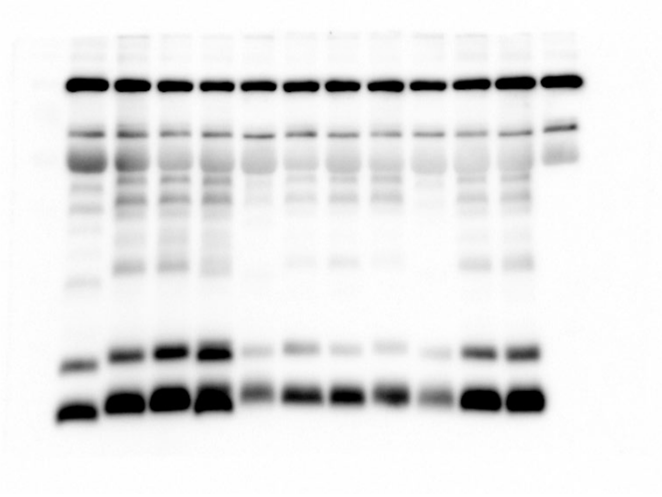

Sample 71-80

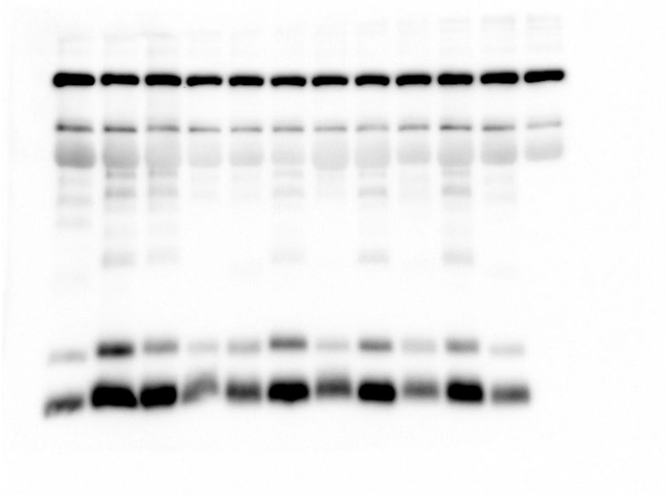

Sample 81-86, 88-91

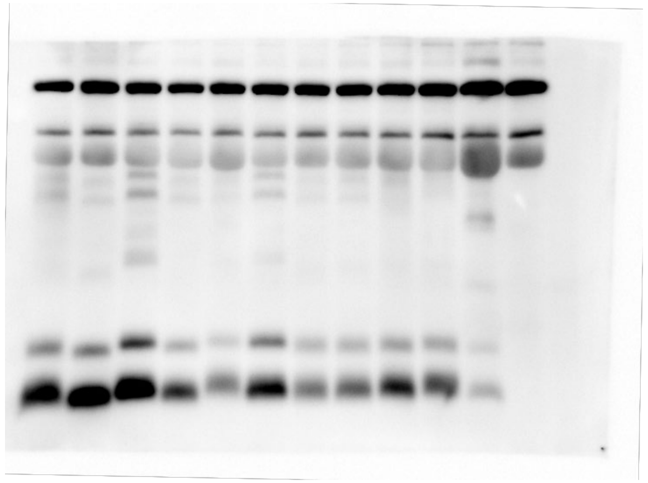

Sample 92-101

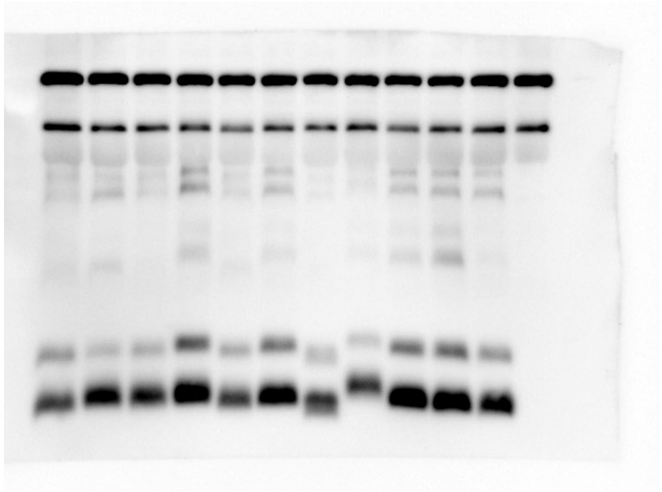

Sample 102-111

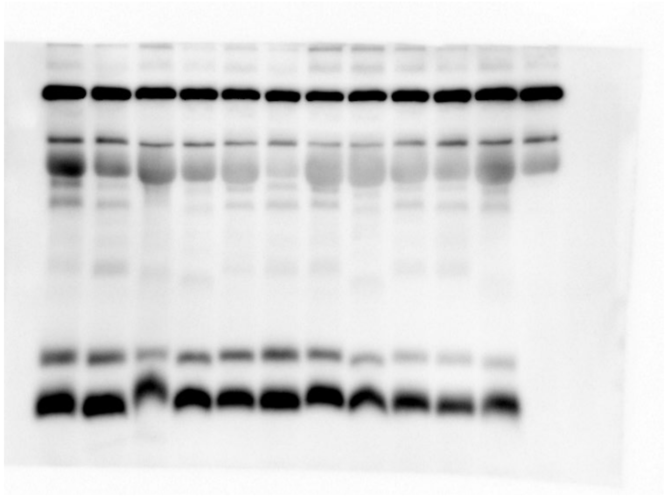

Sample 112-121

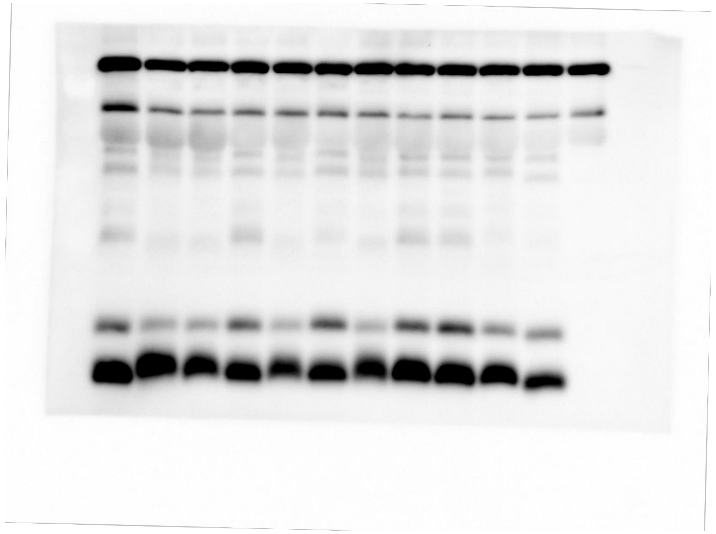

Sample 122-131

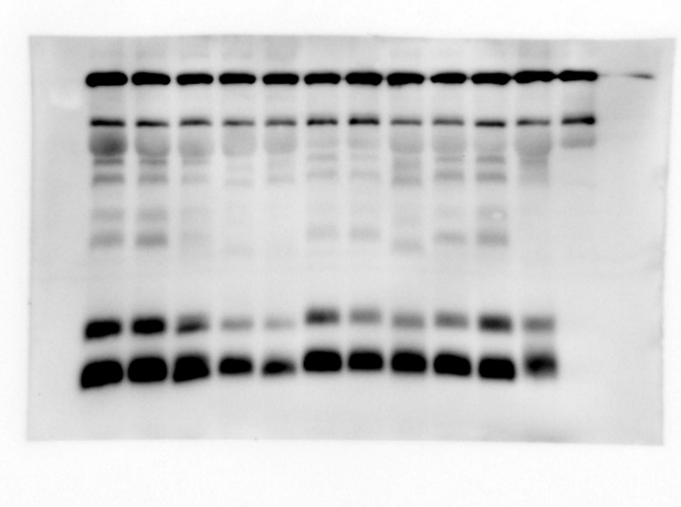

Sample 132-141

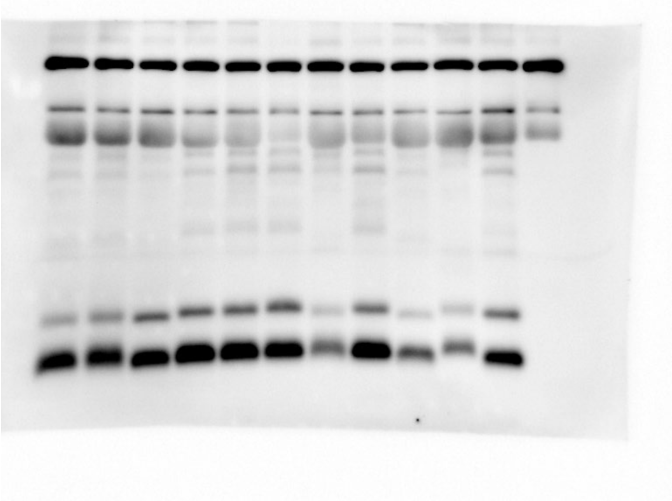

Sample 142-151

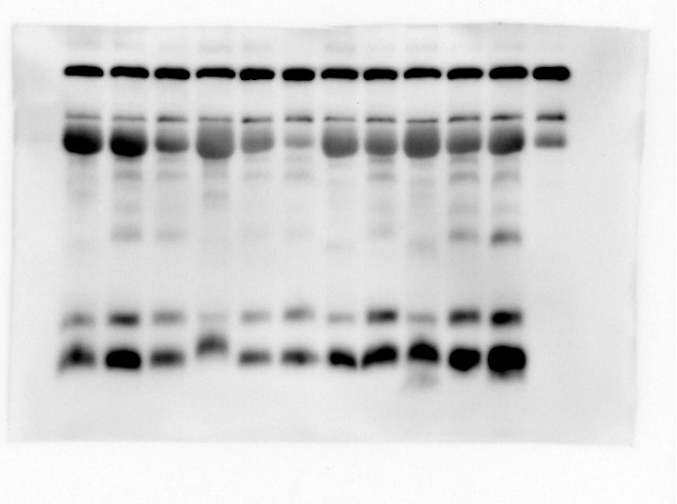

Sample 152-161

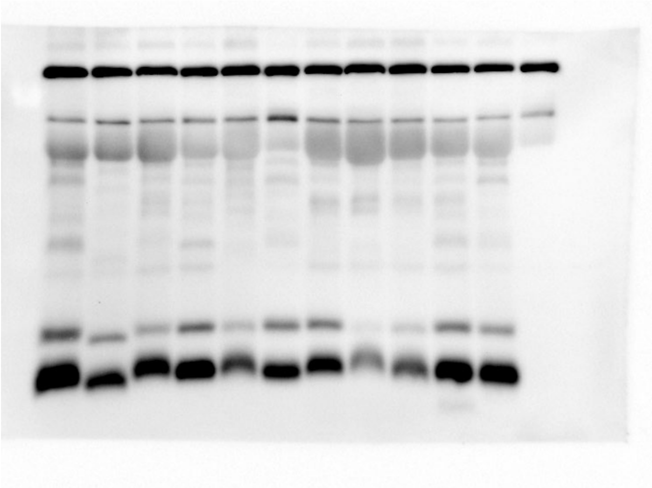

Sample 162-170

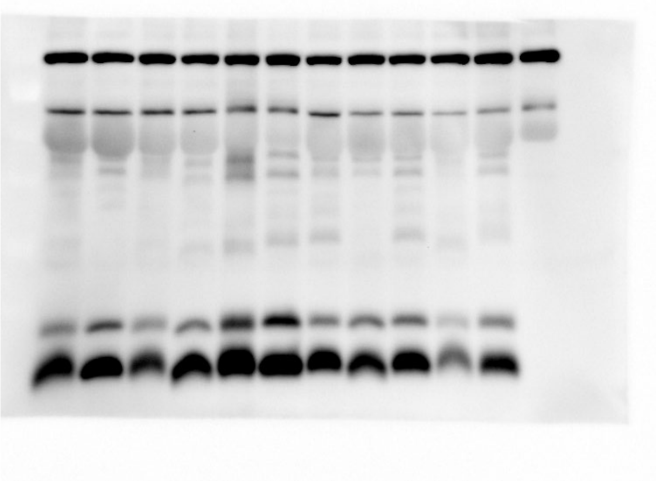

Sample 171-179

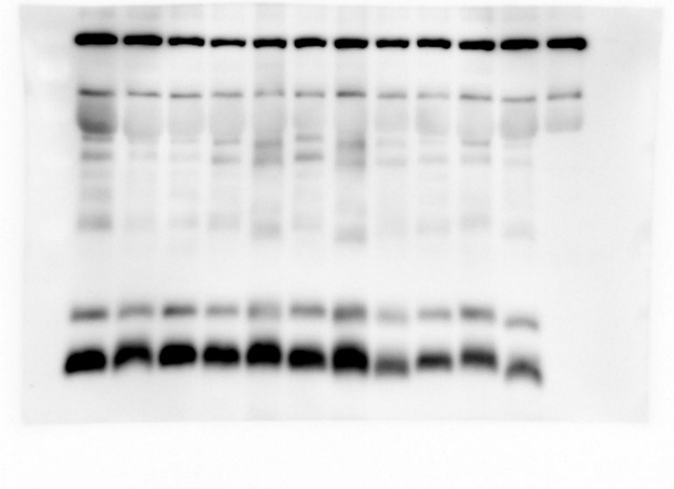

Sample 180-188

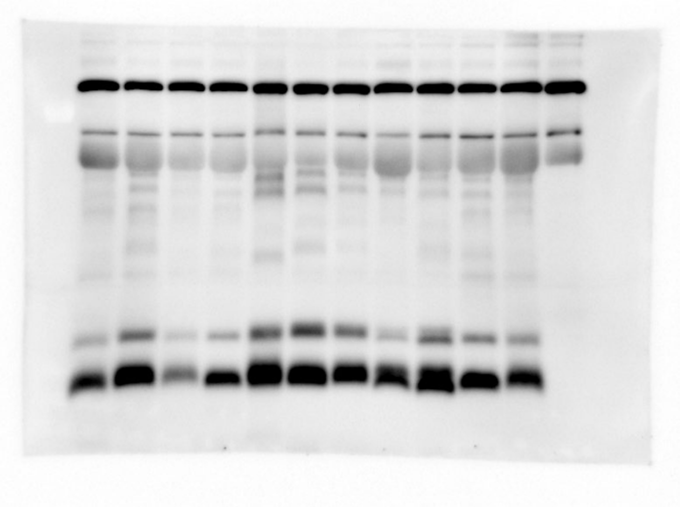

Sample 189-197

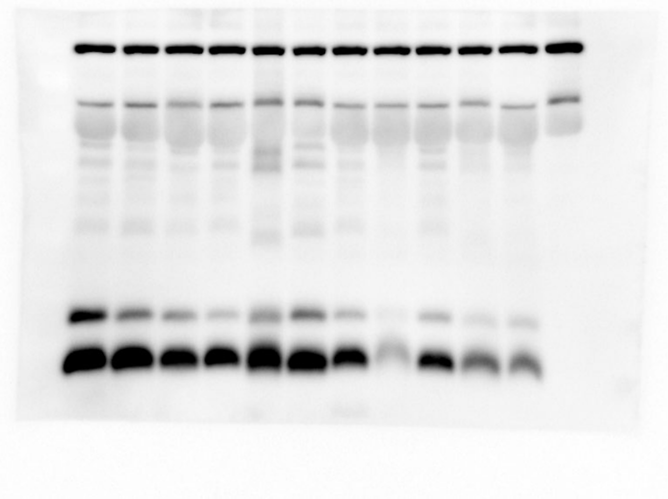

Sample 198-206

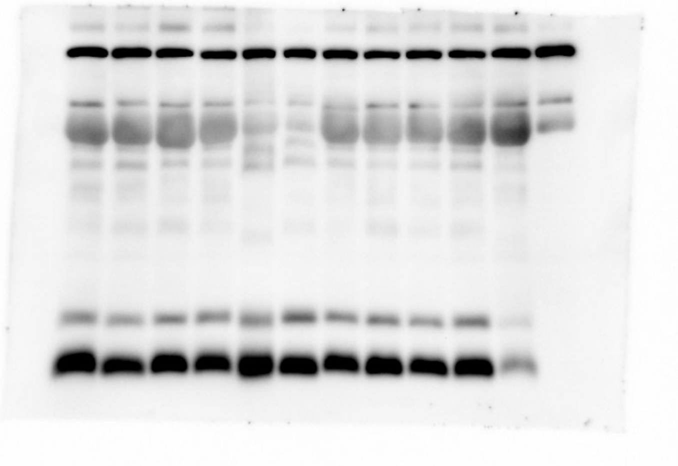

Sample 207-215

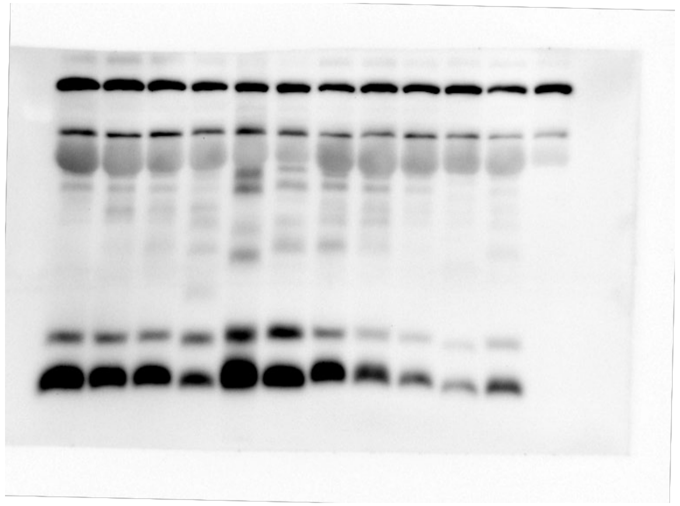

Sample 216-224

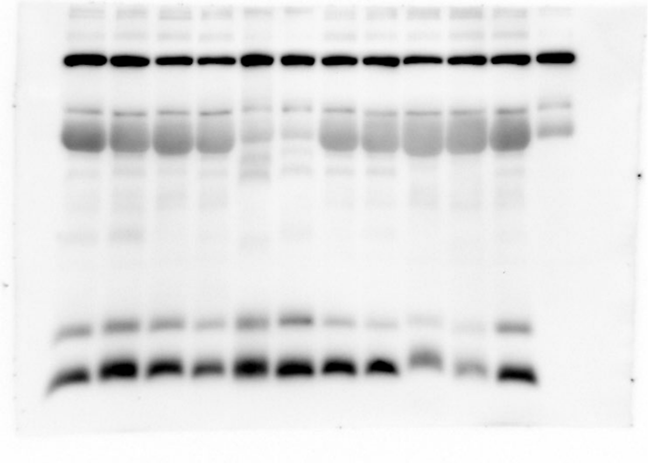

Sample 225-233

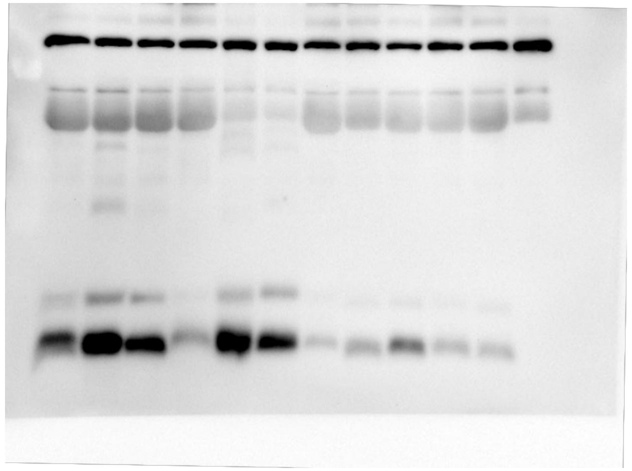

Sample 234-242

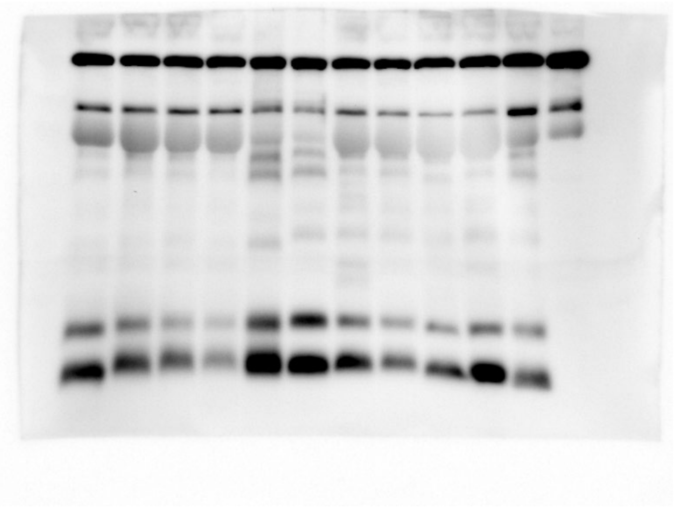

Sample 243-251

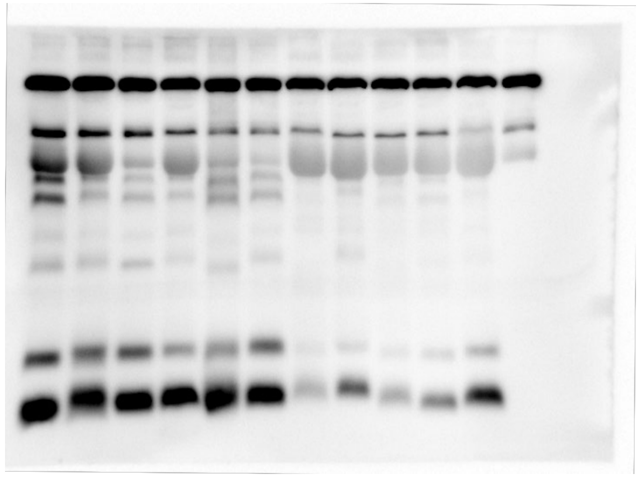

Sample 252-260

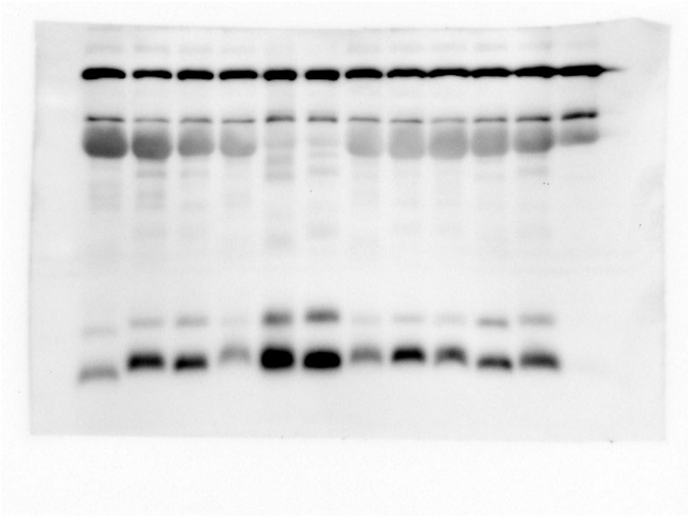

Sample 261-269

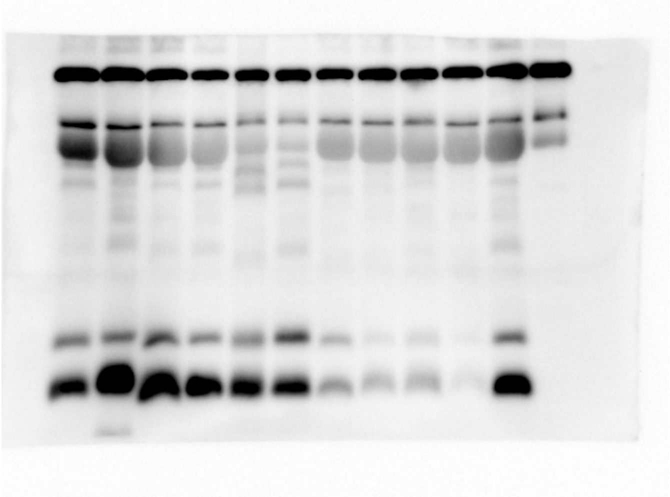

Sample 270-278

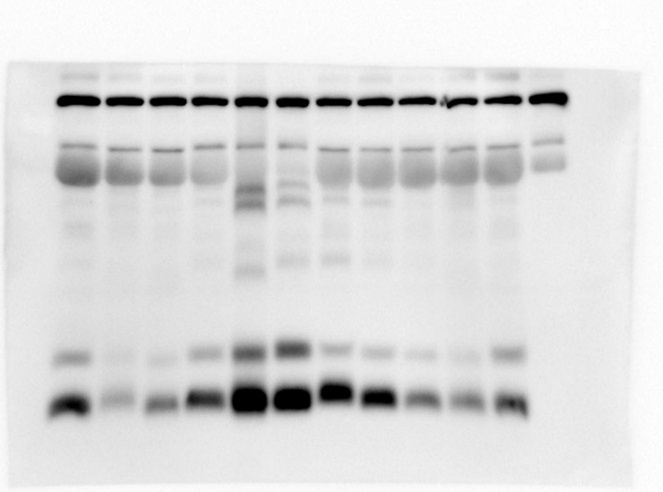

Sample 279-287

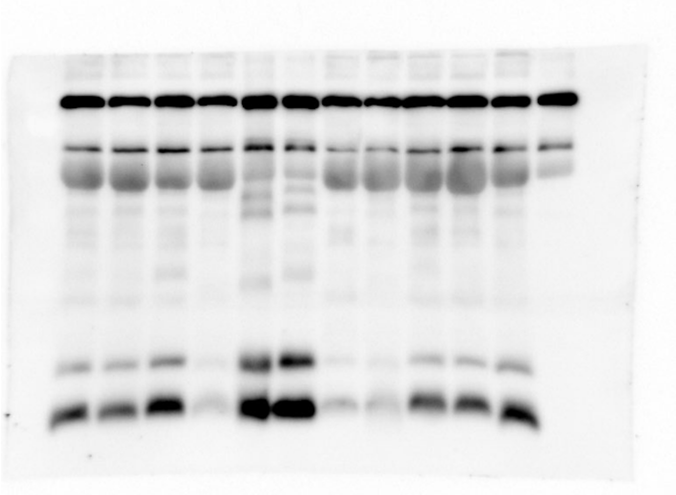

Sample 288-296

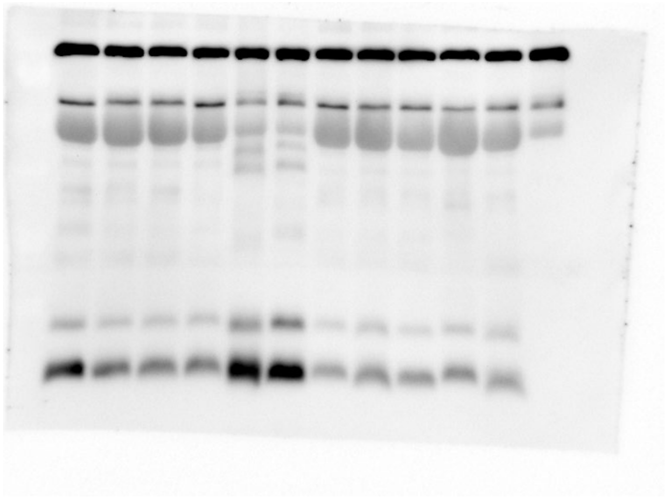

Sample 297-305

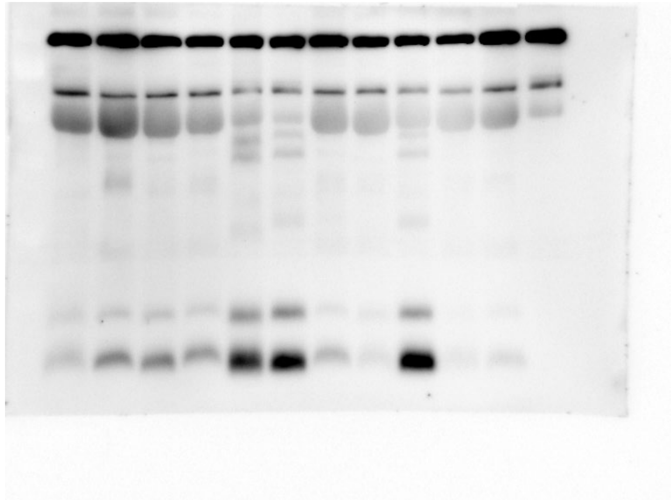

Sample 306-314

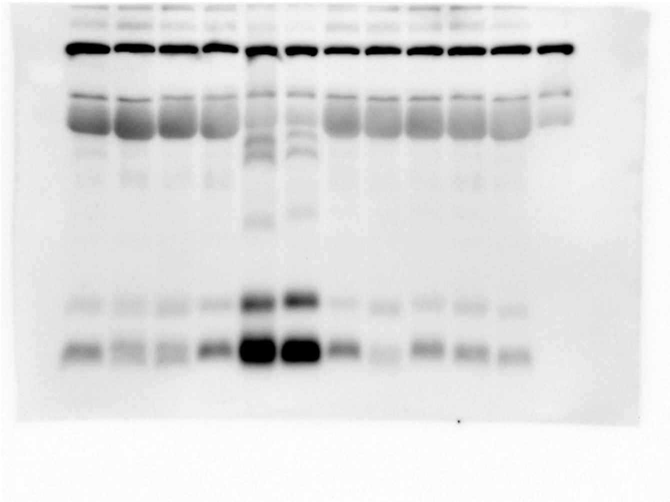

Sample 315-323

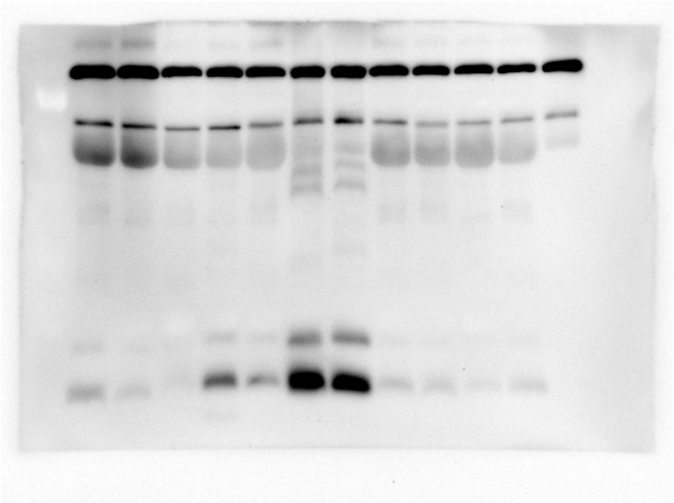

Sample 324-332

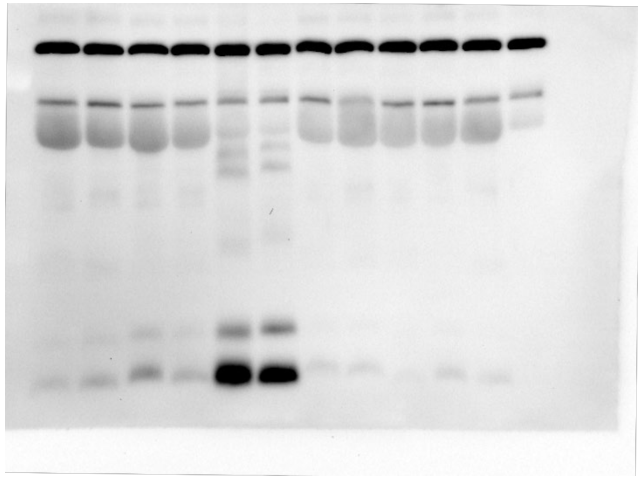

Sample 333-341

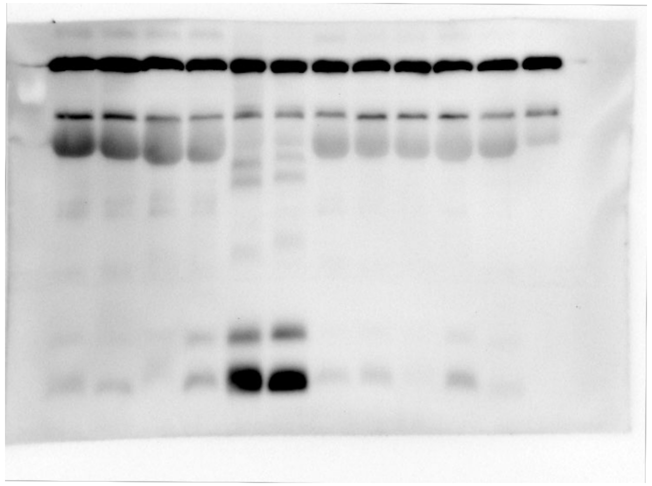

Figure 6C upper

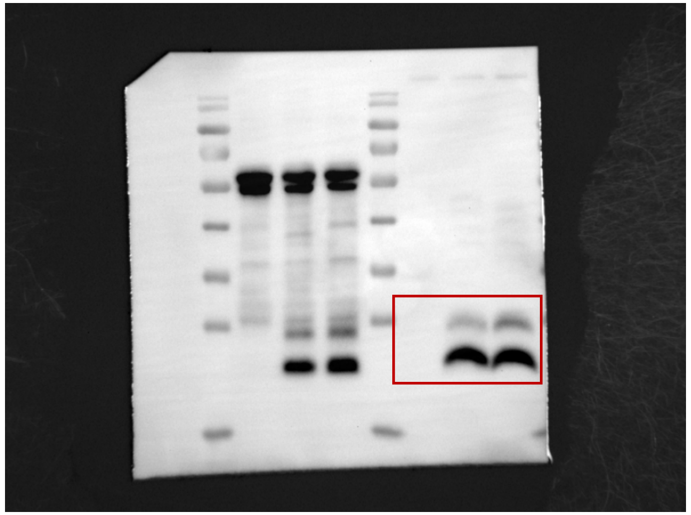

Figure 6C middle

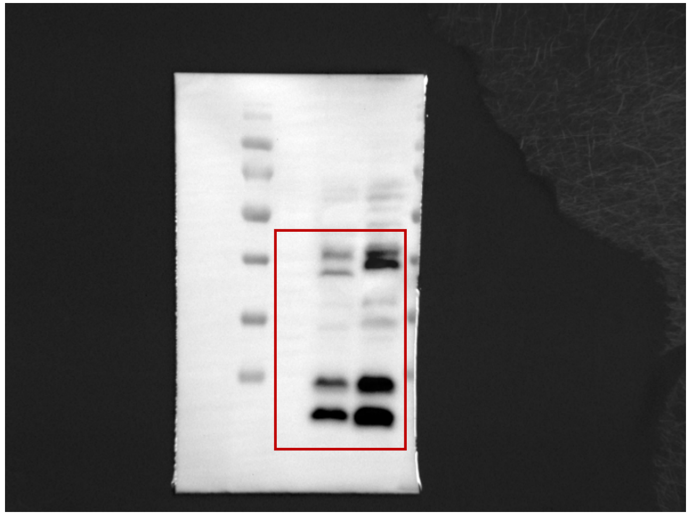

Figure 6C lower

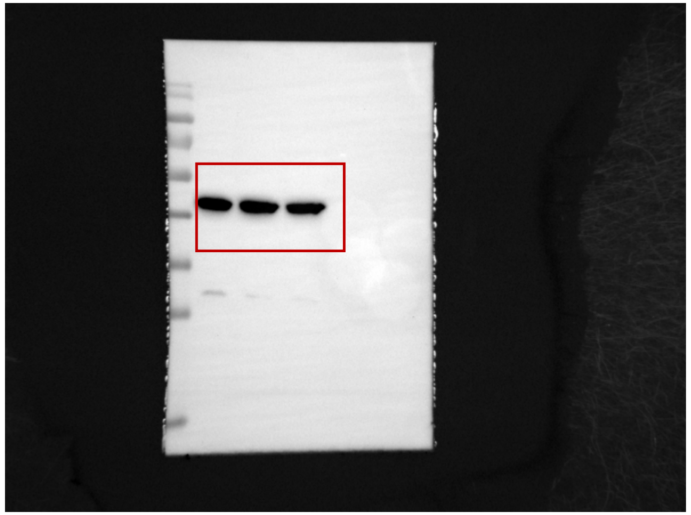

Western blot images used for the **Figure 6C**. The red rectangular box indicates the parts presented in the paper.
