# Supplementary material for: Host sex disparity and viral genotype dependence of the glycosylation level of small Hepatitis B surface protein in patients with HBeAg-positive chronic Hepatitis B
Source: Virol J. 2023 Jul 19;20:159. doi: 10.1186/s12985-023-02096-x (PMC10357594; doi:10.1186/s12985-023-02096-x)
Supplement: Supplementary file 2 — Supplementary Material 2 [file 12985_2023_2096_MOESM2_ESM.docx]

**Supplementary Materials**

**Host sex disparity and viral genotype dependence of the glycosylation level of small hepatitis B surface protein in patients with HBeAg-positive chronic hepatitis B**

Guomin Ou, Chengyu Zhao, Juan Deng, Hui Zhuang*, Kuanhui Xiang*, Tong Li*

Department of Microbiology and Infectious Disease Center, School of Basic Medical Sciences, Peking University Health Science Center, Beijing 100191, China

***Correspondence:**

Hui Zhuang

zhuangbmu@126.com

Kuanhui Xiang

kxiang@bjmu.edu.cn

Tong Li

toglii97@bjmu.edu.cn

**Running Title:** Host sex disparity and viral genotype dependence of the glycosylation level of HBsAg

**Supplementary Methods**

**Preparation of the external control and loading control for WB-HBs assay**

For comparing the levels of SHBs isomers among patients by WB assay, at least one external control (EC) serum is required. The sera S1 to S3 that displayed 6 bands potentially representing ng- and g-L/M/SHBs bands in ordinary WB were preliminarily selected as EC candidates. To verify their sequences encoding HBs without important mutations such as those affecting glycosylation and antigenicity, HBV DNA was extracted from each of 200 μl S1 to S3 serum samples using QIAamp DNA Blood Kit (Qiagen, Hilden, Germany). HBV full genome was amplified with PrimeSTAR Max DNA Polymerase (Takara Bio, United States) according to the well-known method described by Gunther et al[1]. The resulting products were subjected to Sanger sequencing covering preS/S gene in both forward (primers: 5’-gggaacaagagctacagcatg, 5’-tggtggctccagttccgg and 5’-cctcagtccgtttctcctgg) and reverse directions (primers: 5’-ttcctgactgccgattgg, 5’-gagagaagtccaccacgagtc and 5’-ttggtaatagaggtaaaaa) (RuiBiotech, Beijing, China).

The amounts of samples loaded into the SDS-PAGE gel significantly influences the amounts of proteins that can be detected in the blots, which will undoubtedly influence the accuracy of the WB data. To normalize the loading amount in each WB lane, we introduced a recombinant protein His-TF-MHR-AcGFP as a loading control (LC) (**Figure 2A**). MHR including AGL was amplified from aforementioned serum S3 by primers as follows: forward, 5’-ctgttccaggggcccggatccgactaccgaggtatgtggccc; reverse, 5’-gcccttgctcaccatggatccggatgatgggatgggaatacaa. A green fluorescent protein derived from *Aequorea coerulescens* (AcGFP) gene from plasmid pLVX-AcGFP (Takara Bio) was amplified by primers as follows: forward, 5’-atggtgagcaagggc; reverse, 5’-atggagctcgaattctcacttgtacagctca. The resulting MHR and AcGFP fragments were seamlessly cloned into BamHI and EcoRI linearized pCOLD-TF vector (Takara Bio) by one step cloning kit (Vazyme Biotech, China). Trigger factor (TF) located at the upstream of multicloning site of this recombinant plasmid is a kind of *Escherichia coli* (*E. coli)* chaperon genes, which promotes solubility of fusion protein. The 6×hexahistidine (6×His) peptide fused to the N-terminus of TF-MHR-AcGFP allows protein purification by Ni-NTA agarose beads (Qiagen). The predicted molecular weight of recombinant protein His-TF-MHR-AcGFP is 83.8 kD.

The recombinant plasmid pCOLD-His-TF-MHR-AcGFP was introduced into *E. coli* BL21 strain. A single colony of the resulting transformed cells was inoculated into 10 ml LB medium containing 50 μg/ml ampicillin as seed culture and grown overnight at 37℃. The seed culture was inoculated into 500 ml ampicillin-contained LB medium and cultured at 37℃ up to 0.5 of OD_600_. The iso-prophyl-β-D-thiogalactopyranoside was added to ice water pre-cooled culture at a final concentration of 0.4 mM, and further incubation was conducted at 16℃ for 12 h. The induced cells were harvested and centrifuged at 5000 g for 30 min at 4℃. The pellet was re-suspended in binding buffer [20 mM Tris-HCl (pH 8.0), 500 mM NaCl, and 5 mM imidazole] and sonicated on ice using a sonicator (Ningbo Scientz Biotechnology, China). The cell debris were precipitated by centrifugation at 10000 g for 20 min at 4℃ and the supernatant was subjected to a column filled with Ni-NTA agarose beads (Qiagen). After flow through, the beads were washed or eluted with two bed volumes of binding buffer with imidazole concentration of 10, 15, 20, 25 and 400 mM sequentially.

All fractions (4 μl each) were subjected to 10% sodium dodecyl sulfate polyacrylamide gel electrophoresis (SDS-PAGE) and stained with Coomassie blue for visualization. One μl washed fractions were used for detecting the anti-S antibody reactivity by WB assay (description below). The purified LC was quantified by bicinchoninic acid assay kit (Beyotime, China) according to the manufacturer’s instruction.

**Serum samples preparation for quantification of g- and ng-SHBs in WB-HBs assay**

A high level of human serum albumin would limit the loading amount of serum samples (especially those with low HBsAg level) in WB assay, thus the serum samples were pretreated according to the method described by Pfefferkorn et al.[2] with some modifications. In brief, each 50 μl serum sample was diluted in 450 μl phosphate-buffered saline (PBS), then treated with polyethylene glycol 8000 (PEG8000) with a final concentration of 12.5% at 4℃ overnight. After centrifugation of PEG treated serum at 10000 g for 10 min at 4℃, the pellet was washed three times with 12.5% PEG8000 in PBS and resolved in 100 μl PBS. Sample mixture for loading was prepared as follows: each 10 μl of mixture contained 2 μl 5×loading buffer [10% SDS, 0.25% bromophenol blue and 25% glycerol in 250 mM Tris (pH6.8)], the amount of PEG8000 precipitated serum sample or EC corresponding to 0.5 μl or 1 μl of original serum, and 0.25 μg or 0.5 μg of LC. The serum of HBV non-infected individual was used as a negative control (NC) and the EC serum was used for quantifying the relative amount of g- and ng-SHBs of samples in each WB-HBs assay.

**WB-HBs assay, raw data acquisition and novel parameters calculation**

The EC and testing serum samples or HepG2 cells derived samples (the PEG8000 precipitates and cell lysates that would be described in the following section) were loaded and resolved via 12% SDS-PAGE, followed by transferring onto polyvinylidene fluoride (PVDF) membranes. Blotted membranes were blocked using 5% skim milk in Tris-buffered saline with Tween 20 (TBST) for 1 hour at room temperature, followed by hybridizing with horse polyclonal anti-HBs antibody (Abcam, Cambridge, MA, United States) at 1:1000 dilution at 4℃ overnight. The horseradish peroxidase-conjugated anti-horse antibody (Abcam) was used as a secondary antibody at 1:5000 dilution. After incubation for 1 hour at room temperature, the membrane was imaged by ChemiDoc XRS+ System (BioRad) after reaction with SuperSignal West Dura Extended Duration Substrate (Thermo Fisher Scientific, Waltham, MA, United States).

After WB-HBs, the gray values of p24, gp27 and LC bands from EC and testing serum samples were measured by ImageJ software (version: 1.44p) through parameter “IntDen”, which is the product of area and mean gray value of bands on PVDF membranes. Then, the raw data of the target bands (p24 or gp27) from each sample were first normalized by their respective LC. Then, the normalized data of each testing sample were further normalized by EC. This twice normalized value multiplied by 1000 for the sake of convenience was defined as the relative quantity of p24 (ng-SHBs) or gp27 (g-SHBs) of one testing sample. The sum (SHBs=g-SHBs+ng-SHBs) and ratio (g-/ng-SHBs) from testing sample were calculated as well. Thus, four novel parameters reflecting SHBs glycosylation status and relative quantity were introduced in this study (**Figure 2D**).

**In vitro validation of the effect of HBV genotype on SHBs glycosylation**

Plasmid containing 1.3-mer HBV genome of GTC (1.3C) was reported in our previous study[3]. GTB plasmid (a gift from Prof. Yong Lin, Institute for Viral Hepatitis, Chongqing Medical University, China) was reconstructed into 1.3-mer form (1.3B) just like 1.3C, which is described below. Two fragments (F1 and F2) were amplified from the gift plasmid and seamlessly cloned into EcoRI and HindIII linearized vector pGEM3Z by one step cloning kit (Vazyme Biotech). F1 was amplified with following primers: forward, gactcactatagggcgaattcatcctgctttaatgcctttatatgc; reverse, accacgagtctagactctgtggtattgtgaggatt. F2 was amplified with following primers: forward, acagagtctagactcgtggtggacttctctcaatt; reverse, cactatagaatactcaagcttccgatacagagcagaggcgg. To further validation, the 1.0-mer HBV genomes of GTB and GTC were also amplified from the sera of CHB patients. The 1.3-mer HBV plasmids named as 1.3b and 1.3c were constructed using the same protocol[3]. The amino acid differences in 1.3b SHBs sequences are Y76C, F161Y and I198M compared to 1.3B, while in 1.3c are S3N, A47T, L49P, P62L and L213I compared to 1.3C.

HepG2 cells (a gift from Prof. Charles M. Rice, Rockefeller University, United States) were maintained in Dulbecco’s modified Eagle’s medium (DMEM) with 10% fetal bovine serum (FBS), 100 IU/ml penicillin, and 100 mg/ml streptomycin in collagen I-precoated plates in a 5% CO_2_ incubator at 37℃. HepG2 cells of 5×10^4^/well in 24-well plate or 3×10^5^/well in 6-well plate were transiently transfected with 0.5 μg or 2 μg plasmids, respectively. Transfection was carried out using the Lipofectamine 2000 (Thermo Fisher Scientific) according to the manufacturer’s instruction. Two days after transfection, the supernatants of 24-well plates were harvested for detecting HBeAg level, a marker of HBV replication. The HBeAg (quantification range: 0.1-200 PEI U/ml) was quantified by chemiluminescence immunoassay (CLIA) kits (Autobio diagnostics, Zhengzhou, China) according to the manufacturer’s instruction. Precipitation of HBV associated particles by adding PEG8000 to 500 μl supernatants of 6-well plate to a final concentration of 12.5% was followed by incubation at 4℃ overnight. The precipitates were collected by centrifugation at 10000 g for 10 min at 4℃ and dissolved in 100 μl PBS. HepG2 cells were lysed with 200 μl RIPA buffer. HepG2 cells derived samples (the PEG8000 precipitates and cell lysates) were analyzed by WB assay.

**Supplementary Tables**

**Supplementary Table S1. The sequence analysis of preS/S gene amplified from 3 serum samples of S1 to S3**

| **Serum** | **Patient ID** | **Genotype** | **HBV DNA**  **(log_10_ IU/ml)** | **HBsAg**  **(log_10_ IU/ml)** | **Length (AA)** | | |  | **Detection of mutation** | | |  |
| --- | --- | --- | --- | --- | --- | --- | --- | --- | --- | --- | --- | --- |
|  |  |  |  |  | **preS1** | **preS2** | **S** |  | **N-glycosylation site** | **O-glycosylation site** | **Antigenicity related sites*** | |
| S1 | A00016 | C | 8.61 | 4.70 | 119 | 55 | 226 |  | No | No | No | |
| S2 | A01135 | C | 8.29 | 4.76 | 119 | 55 | 226 |  | No | No | No | |
| S3 | A01414 | C | 8.85 | 4.78 | 119 | 55 | 226 |  | No | No | No | |

AA, amino acid; HBsAg, hepatitis B surface antigen; HBV, hepatitis B virus; *****, the SHBs sequences of 3 serum samples were compared with the SHBs consensus from our previous report[4] and the mutations in classic antigenicity related sites described in papers[5–7] were not detected

**Supplementary Table S2. Intra- (12-fold replicates in one run) and inter- (one sample in 15 runs) assay coefficient of variation of the tested samples**

| **Parameter** | **Intra-assay (N=12)** | |  | | **Inter-assay (N=15)** | |
| --- | --- | --- | --- | --- | --- | --- |
|  | **Median (range)** | **Coefficient of variation (%)** | |  | **Median (range)** | **Coefficient of variation (%)** |
| g-SHBs (log_10_) | 2.95 (2.88-3.13) | 2.87 | |  | 2.57 (2.47-2.70) | 2.39 |
| ng-SHBs (log_10_) | 3.28 (3.21-3.33) | 1.20 | |  | 3.04 (2.93-3.08) | 1.39 |

g-, glycosylated; ng-, non-glycosylated; SHBs, small hepatitis B surface protein

**Supplementary Figures**


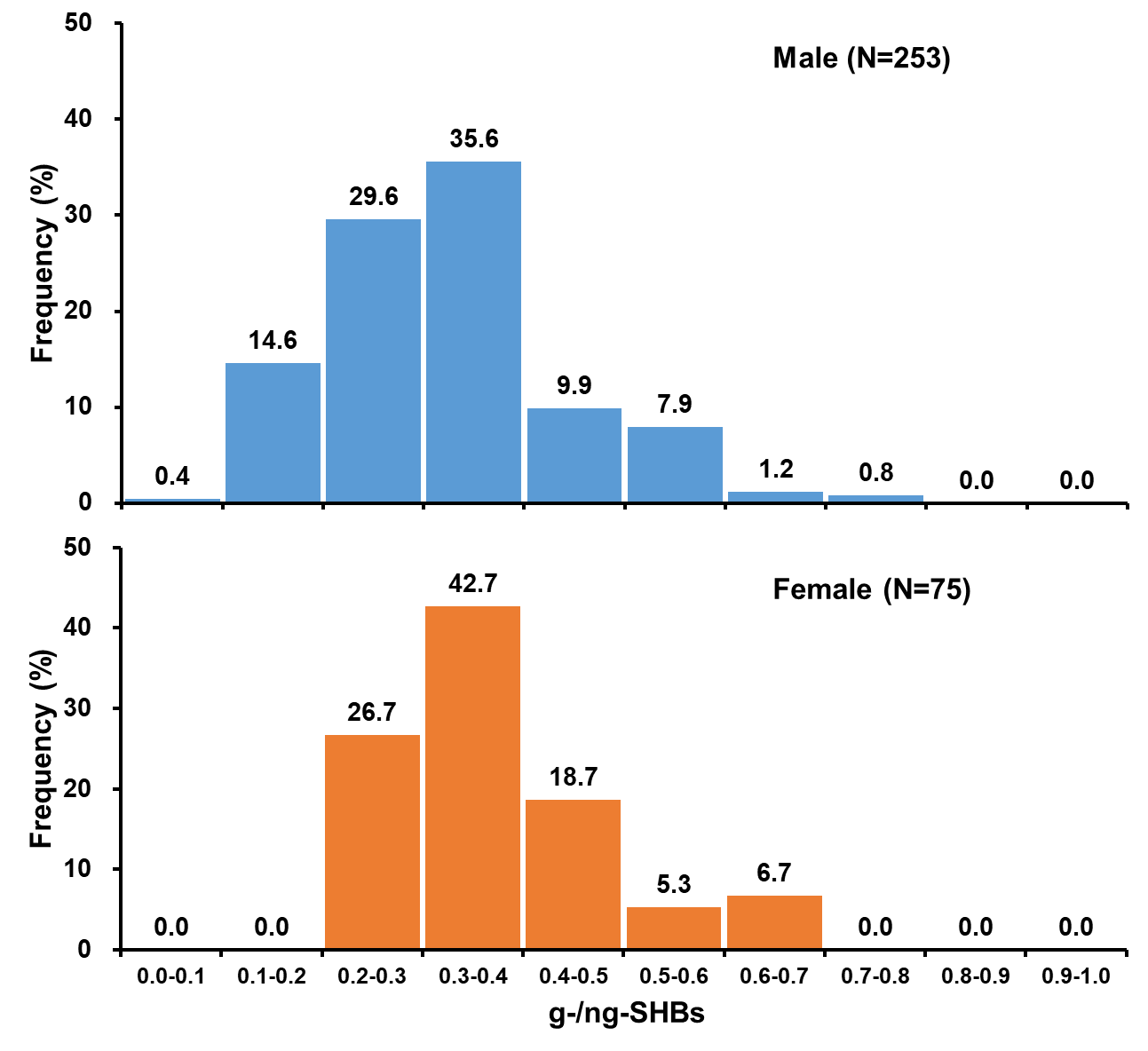


**Supplementary Figure S1**. The frequency distribution of g-/ng-SHBs ratios in male and female patient groups. The frequencies of g-/ng-SHBs ratios were indicated above the column


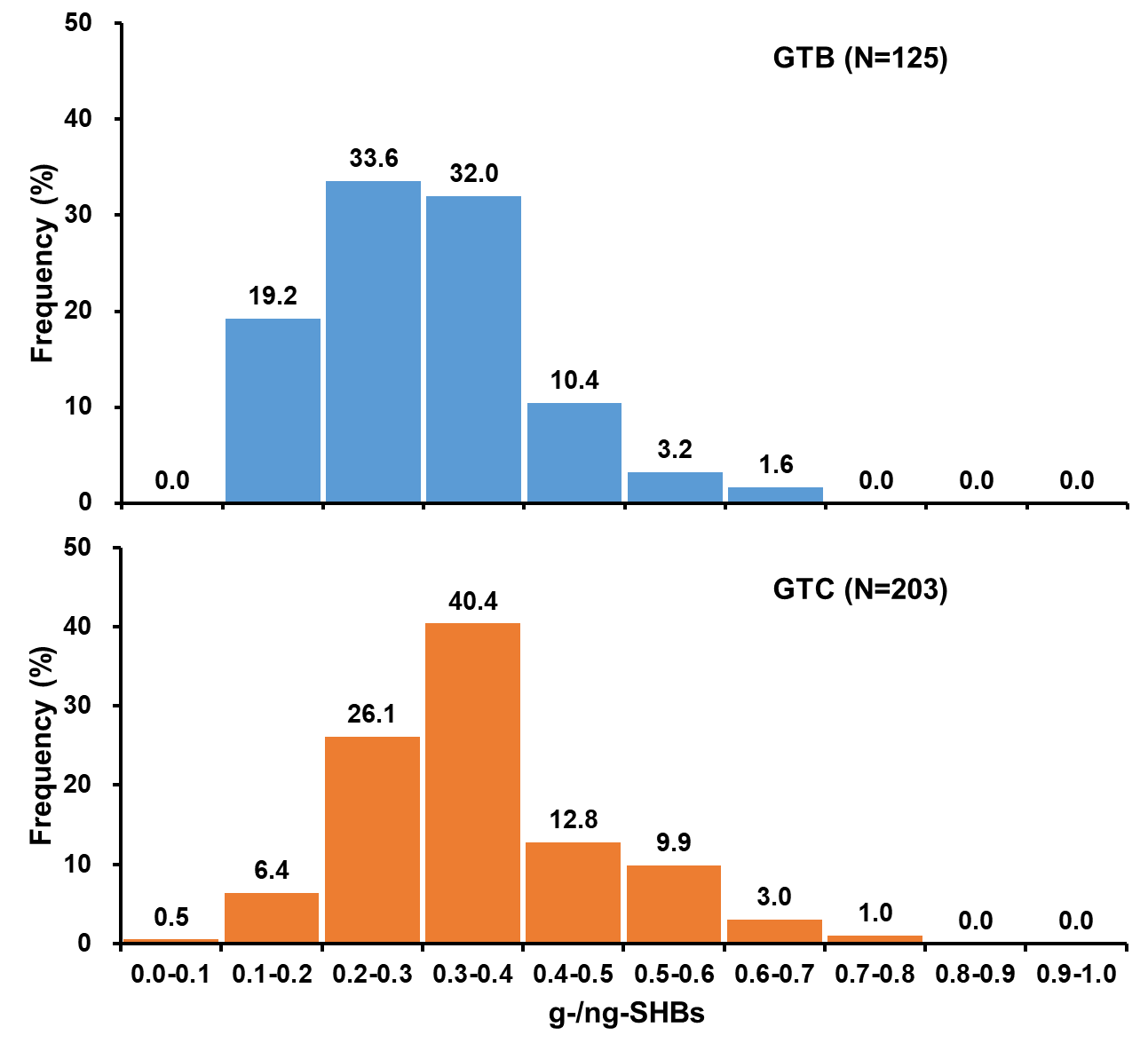


**Supplementary Figure S2**. The frequency distribution of g-/ng-SHBs ratios in patients with GTB and GTC. The frequencies of g-/ng-SHBs ratios were indicated above the column

**
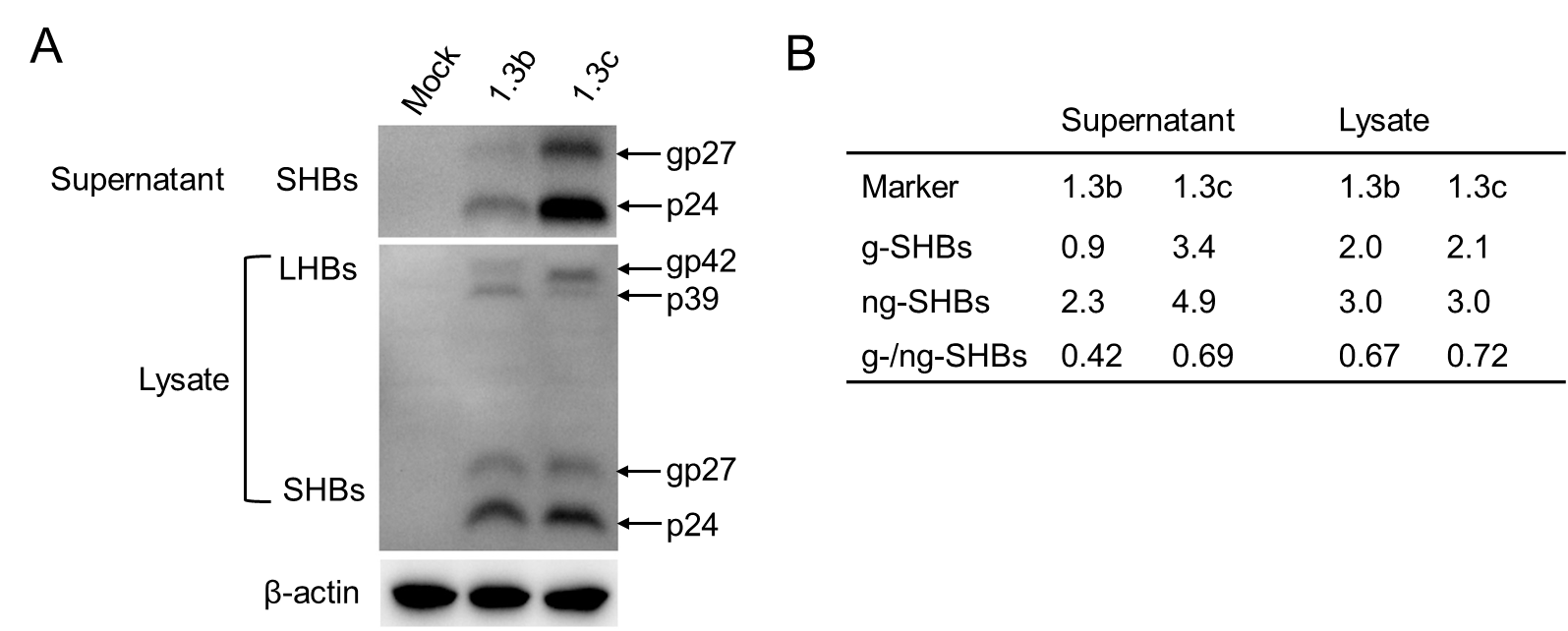
**

**Supplementary Figure S3**. The level of g-/ng-SHBs ratio in HBV genotype (GT) C is higher than that in GTB in vitro tested by two other HBV plasmids. 1.3-mer HBV genomes of GTB (1.3b) and GTC (1.3c) from patient sera were cloned into pGEM3Z plasmid according to method[3]. HepG2 cells were transfected with 1.3b, 1.3c or pGEM3Z mock vector in 6-well plate. At 48 h post transfection, precipitation of HBV associated particles by adding polyethylene glycol 8000 to 500 μl supernatants from 6-well plate to a final concentration of 12.5% was followed by incubation at 4℃ for overnight. The precipitates were collected by centrifugation at 10000 g for 10 min and dissolved in 100 μl PBS. HepG2 cells of 6-well plate were lysed with 200 μl RIPA buffer. (A) The dissolved precipitates and cell lysates were subjected to Western blot assay. (B) The IntDen values of g- and ng- SHBs in supernatants and lysates were measured by ImageJ software and the levels of g-/ng-SHB ratio were calculated

**References**

1. Gunther S, Li BC, Miska S, Kruger DH, Meisel H, Will H. A novel method for efficient amplification of whole hepatitis B virus genomes permits rapid functional analysis and reveals deletion mutants in immunosuppressed patients. J Virol. 1995;69:5437–44.

2. Pfefferkorn M, Bohm S, Schott T, Deichsel D, Bremer CM, Schroder K, et al. Quantification of large and middle proteins of hepatitis B virus surface antigen (HBsAg) as a novel tool for the identification of inactive HBV carriers. Gut. 2018;67:2045–53.

3. Ou G, He L, Wang L, Song J, Lai X, Tian X, et al. The genotype (A to H) dependent N-terminal sequence of HBV large surface protein affects viral replication, secretion and infectivity. Front Microbiol. 2021;12:687785.

4. Ding H, Liu B, Zhao C, Yang J, Yan C, Yan L, et al. Amino acid similarities and divergences in the small surface proteins of genotype C hepatitis B viruses between nucleos(t)ide analogue-naïve and lamivudine-treated patients with chronic hepatitis B. Antiviral Res. 2014;102:29–34.

5. Tong S, Revill P. Overview of hepatitis B viral replication and genetic variability. J Hepatol. 2016;64:S4–16.

6. Echevarria JM, Avellon A. Hepatitis B virus genetic diversity. J Med Virol. 2006;78:S36–42.

7. Kwei K, Tang X, Lok AS, Sureau C, Garcia T, Li J, et al. Impaired virion secretion by hepatitis B virus immune escape mutants and its rescue by wild-type envelope proteins or a second-site mutation. J Virol. 2013;87:2352–7.
